# Genome-wide identification and characterization of polycomb repressive complex 2 core components in upland cotton (*Gossypium hirsutum* L.)

**DOI:** 10.1186/s12870-023-04075-4

**Published:** 2023-02-01

**Authors:** Kai Cheng, Cangbao Lei, Siyuan Zhang, Qiao Zheng, Chunyan Wei, Weiyi Huang, Minghui Xing, Junli Zhang, Xiangyu Zhang, Xiao Zhang

**Affiliations:** State Key Laboratory of Cotton Biology, School of Life Sciences, Henan University, 475001 Kaifeng, China

**Keywords:** PRC2, Genome-wide identification, Gene expression, Upland cotton

## Abstract

**Background:**

The evolutionarily conserved Polycomb Repressive Complex 2 (PRC2) plays a vital role in epigenetic gene repression by depositing tri-methylation on lysine residue K27 of histone H3 (H3K27me3) at the target loci, thus participating in diverse biological processes. However, few reports about PRC2 are available in plant species with large and complicated genomes, like cotton.

**Results:**

Here, we performed a genome-wide identification and comprehensive analysis of cotton PRC2 core components, especially in upland cotton (*Gossypium hirsutum*). Firstly, a total of 8 and 16 PRC2 core components were identified in diploid and tetraploid cotton species, respectively. These components were classified into four groups, E(z), Su(z)12, ESC and p55, and the members in the same group displayed good collinearity, similar gene structure and domain organization. Next, we cloned *G. hirsutum* PRC2 (GhPRC2) core components, and found that most of GhPRC2 proteins were localized in the nucleus, and interacted with each other to form multi-subunit complexes. Moreover, we analyzed the expression profile of GhPRC2 genes. The transcriptome data and quantitative real-time PCR (qRT-PCR) assays indicated that GhPRC2 genes were ubiquitously but differentially expressed in various tissues, with high expression levels in reproductive organs like petals, stamens and pistils. And the expressions of several GhPRC2 genes, especially E(z) group genes, were responsive to various abiotic and biotic stresses, including drought, salinity, extreme temperature, and *Verticillium dahliae* (*Vd*) infection.

**Conclusion:**

We identified PRC2 core components in upland cotton, and systematically investigated their classifications, phylogenetic and synteny relationships, gene structures, domain organizations, subcellular localizations, protein interactions, tissue-specific and stresses-responsive expression patterns. Our results will provide insights into the evolution and composition of cotton PRC2, and lay the foundation for further investigation of their biological functions and regulatory mechanisms.

**Supplementary Information:**

The online version contains supplementary material available at 10.1186/s12870-023-04075-4.

## Background

The precisely spatio-temporal regulation of gene transcription is critical for development and environmental response in eukaryotes, including plants. Among the large number of transcriptional regulators, Polycomb group (PcG) proteins play vital roles in epigenetic transcription silence by establishing and maintaining a repressed chromatin state at the target loci [1–3]. PcG proteins were originally identified as regulators of homeobox (*HOX*) genes expression during segmentation in *Drosophila* [4], and found in many other species thereafter. PcG proteins can form two major multiprotein complexes, Polycomb Repressive Complex 1 (PRC1), which catalyzes the ubiquitylation of histone H2A Lys119 in animals and Lys121 in plants (H2AK119/121ub) [5, 6], and PRC2, which mediates histone H3 lysine 27 trimethylation (H3K27me3) [7–9]. Several other PcG complexes were also reported, for instance, the DNA binding of Pho-repressive complex (PhoRC) is critical for PRC1 targeting to Polycomb response elements (PREs) [10, 11], whereas polycomb-like PRC2 (Pcl-PRC2) is needed to generate high levels of H3K27me3 at target genes in *Drosophila* [12]. A hierarchical recruitment model has been used to explain PcG-mediated transcription repression for a long time: PRC2 binds to target genes and incorporates H3K27me3, and then PRC1 is recruited and mediates H2AK119/121ub to maintain the stable repressive chromatin state [13]. However, recent studies have revealed that PRC1 activity and H2AK119/12ub marking are independent of PRC2 activity, and are required for PRC2 recruiting and H3K27me3 deposition, which virtually overturns the classic hierarchy [13, 14].

*Drosophila* PRC2 is composed of four core components: the histone methyltransferase Enhancer of zeste [E(z)], Suppressor of zeste 12 [Su(z)12], Extra sex combs (ESC), and Nucleosome remodeling factor 55 kDa (Nurf55/p55). Likewise, plant PRC2 complexes also consist of the four conserved subunits, with more members in each subunit [1, 2]. In *Arabidopsis*, three E(z) homologs [CURLY LEAF (CLF), SWINGER (SWN) and MEDEA (MEA)], three Su(z)12 homologs [FERTILIZATION INDEPENDENT SEED2 (FIS2), EMBRYONIC FLOWER2 (EMF2) and REDUCED VERNALIZATION RESPONSE2 (VRN2)], five p55-like proteins [MULTICOPY SUPPRESSOR OF IRA1-5 (MSI1-5)], and a single ESC copy FERTILIZATION INDEPENDENT ENDOSPERM (FIE) have been identified up to now. The duplication enables alternative combinations of these four subunits to form at least three distinct PRC2 complexes, named FIS-PRC2 (FIS2, MEA, FIE, MSI1), EMF2-PRC2 (EMF2, CLF/SWN, FIE, MSI1), and VRN2-PRC2 (VRN2, CLF/SWN, FIE, MSI1) [1–3, 15, 16]. PRC2 components have been also identified in other plant species, including rice [17], maize [18, 19], green lineage [20], *Brachypodium distachyon* [21], barley [22], bread wheat [23] and *Medicago truncatula* [24]. Notably, the composition of PRC2 complexes displays considerable variability in different species. For example, the equivalents of MEA and FIS2, two core components of *Arabidopsis* FIS-PRC2, as well as that of VRN2, an essential subunit of *Arabidopsis* VRN2-PRC2, are absent in cereals [17–19, 22, 23], whereas the counterpart of FIE and EMF2, the single ESC homolog and one of the three Su(z)12 homologs in *Arabidopsis* respectively, are duplicated in both rice and maize [17–19].

A large number of studies have highlighted the essential roles of PRC2 in the repression of target genes during plant growth and development. In *Arabidopsis*, FIS-, EMF2-, and VRN2-PRC2 complexes regulate diverse biological processes in a distinct but interweaved manner [1–3, 15, 16]. FIS-PRC2 is required to prevent endosperm development in the absence of fertilization, partially though incorporating H3K27me3 marks on several imprinted genes such as *PHE1* [25], *AGL62* [26], and a set of C2 type I MADS-box genes [27]. Mutation of FIS-PRC2 components, such as MEA and FIS2, causes the initiation of autonomous endosperm development without fertilization and the production of autonomous seeds derived from the female gametophytic central cell [28]. SWN has partially overlapping functions with MEA in seed development, and *swn mea* double mutants display a more severe phenotype [29]. EMF2-PRC2 is critical for developmental phase transitions, from the embryonic to vegetative and the vegetative to reproductive. On one hand, EMF2-PRC2 elevates H3K27me3 accumulation at seed maturation genes such as *DOG1*, *ABI3*, *LEC1/2* and *FUS3*, thus promoting seedling development [30]; on the other, EMF2-PRC2 represses the expression of *FT* and floral homeotic genes like *AG* to prevent premature flowering by regulation H3K27me3 profile at the relevant loci [31]. It is worth noting that the regulatory functions of EMF2-PRC2 during these transitions require PRC1 activity and H2AK121ub marking [32], and the coordination of other epigenetic regulators, including TrxG proteins ATX1 and ULT1 [33], chromatin remodelers PKL [34] and BRM [35]. VRN2-PRC2 controls the floral transition and reproductive development, during which VRN2-PRC2, in conjunction with three PHD finger proteins, VRN5, VIN3, and VEL1, epigenetically silences *FLC* transcription by incorporating repressive H3K27me3 marks at the *FLC* loci, thus relieving the inhibition on *FT* expression and triggering flowering [36]. The induction of flowering is also implicated with the repression of *FLC* relatives *MAFs*, *SVP* and *VIN3* [37]. Interestingly, MSI1, the p55 homologs present in all three PRC2 complexes, is reported as a multi-faceted regulators of the flowering time. Besides as the preventer of premature flowering during vegetative development, and the inducer of vernalization-dependent flowering described previously, MSI1 also acts upstream of the *CO*-*FT* pathway to promote photoperiodic flowering via an unclear mechanism [38]. MSI1 also physically interacts with a histone deacetylase HDA6, and they interdependently regulates the profiles of H3ac and H3K27me3 modification at *FLC*, *MAF4*, and *MAF5* loci, thus fine-tuning flowering time [39].

Emerging evidences have uncovered the important roles of PRC2 components on plant adaption to the environmental stimuli. The phytohormone abscisic acid (ABA) is essential for plant development and abiotic stress responses. Two core enzymatic subunits, CLF and SWN, promote H3K27me3 deposition at ABA-induced senescence-associated genes and repress their expression, thereby participating ABA-triggered senescence, which may contribute to enhancing stress tolerance [40]. MSI1 functions in a HDA19-containing complex to fine-tune ABA signaling and salt stress response though modulating the H3K9ac level at ABA receptor genes, thus affecting their expression levels [41]. CLF and its product H3K27me3 marks at *LTP3*, *LTP4*, *HIPP2.2*, *RAB18*, and *RD29B*, are also required for the memory of repetitious dehydration stress response [42]. A recent study reported that CLF concomitantly represses *SEPALLATA3* and activates *Octadecanoid-responsive Arabidopsis 59* (*ORA59*), thus regulating the leaf immunity to *Colletotrichum fungi* [43]. However, the roles of PRC2 and its components in aspects beyond growth and development remain largely unknown.

Cotton (Gossypium spp.) is one of the most important economic crops worldwide as sources of natural fibers as well as edible oil and protein. More than 50 cotton species are distributed in the tropic and subtropic areas. Among the current cultivars, the upland cotton, allotetraploid *G. hirsutum*, provides more than 90% of raw materials for cotton commercial production [44]. Despite the large scale and highly subgenomic homology, the high-quality genome sequencing and assembly of more and more cotton species have been completed, including diploid cottons *G. raimondii*, *G. arboreum*, and *Gossypium austral*, and allotetraploid cottons *G. hirsutumtm* and *G. barbadense* [45]. The great improvement on cotton genome research enables the genome-wide identification and systematic analysis of many gene families related to the cultivation traits. Nevertheless, only a few of epigenetic regulators have been reported in cotton. For example, histone deacetylase GhHDA5 is involved in fiber initiation by removing H3K9ac marks at fiber initiation-specific genes and modulating their expression in *G. hirsutum* [46]. A recent study reported that a cotton PRC2 component, GhEMF2, coming from an earliness-related QTL, represses the floral transition by regulating the expression of the positive floral regulators *GhAGL6*, *GhFT* and *GhAP1* [47, 48]. However, no systemic identification and analysis of cotton PRC2 have been reported.

In this study, we identified PRC2 core components in three cotton species and investigated their phylogenetic and synteny relationships. We also cloned and characterized PRC2 components from *G. hirsutum*, including the gene and protein structures, subcellular localizations, protein–protein interaction patterns, and expression profiles. Our results may provide useful resource for further researches about the biological roles and regulatory mechanisms of cotton PRC2.

## Results

### Identification of cotton PRC2 core components

To identify cotton PRC2 core components, a BLASTP search using *Arabidopsis* PRC2 proteins as queries was employed against the cotton genome data. A total of 8, 8 and 16 PRC2 proteins were identified in *G. arboreum*, *G. raimondii*, and *G. hirsutum*, respectively. These proteins were renamed after their *Arabidopsis* homologs, and the “A” and “D” were appended to GhPRC2 components to distinguish the At- and Dt-subgenomes. All of cotton PRC2 components displayed high identities with their *Arabidopsis* orthologs (Additional file 1: Table S1). The diploid *G. arboretum* and *G. raimondii* possessed the same number of PRC2 components with *Arabidopsis*, and the tetraploid *G. hirsutum* harbored twice as many PRC2 proteins as the diploid species (Table 1), indicating that PRC2 is highly conserved in the process of polyploidy in cotton species. It is worth noting that two CLF and EMF2 homologs were identified in *G. arboretum* and *G. raimondii*, and four in *G. hirsutum*, suggesting a gene duplication events during the course of cotton evolution. No MEA or FIS2 orthologs were found in three cotton species, which were also absent in cereals [17–19, 22, 23, 49].

**Table 1:** PRC2 core components identified in three cotton species

Subunit	Name^a^	Gene ID	Genome localization	CDS length (bp)	No. of exons	Protein length (aa)	MW (kDa)	pI	Charge	GRAVY
**E(z)**	GaCLF-1	Ga10G2073	Chr10:110,747,653–110,755,310	2763	17	920	103.329	8.608	28.5	-0.81
	GaCLF-2	Ga11G1924	Chr11:89,908,029–89,918,634	2769	17	922	103.045	8.431	25.5	-0.722
	GaEZA1	Ga12G1657	Chr12:25,632,777–25,639,592	2682	17	893	99.665	7.591	13.5	-0.77
	GrCLF-1	Gorai.011G106100	Chr11:12,348,918–12,357,283	2760	17	919	103.374	8.728	32	-0.806
	GrCLF-2	Gorai.007G215200	Chr07:23,786,559–23,797,878	2796	17	931	104.551	8.488	28	-0.672
	GrEZA1	Gorai.008G139100	Chr08:38,989,212–38,996,424	2661	17	886	98.898	7.399	10	-0.778
	GhCLF-1A	Gh_A10G0823	A10:17,007,592–17,015,253	2763	17	920	103.344	8.648	29.5	-0.818
	GhCLF-1D	Gh_D10G0937	D10:12,479,849–12,487,638	2793	17	930	104.683	8.624	29.5	-0.782
	GhCLF-2A	Gh_A11G1788	A11:32,190,386–32,201,011	2769	17	922	103.047	8.412	25.5	-0.719
	GhCLF-2D	Gh_D11G1949	D11:24,534,860–24,545,438	2766	17	921	102.801	8.471	27	-0.733
	GhEZA1-A	Gh_A12G1126	A12:64,669,626–64,676,441	2661	17	886	98.817	7.488	11	-0.773
	GhEZA1-D	Gh_D12G1255	D12:40,655,858–40,662,659	2685	17	894	99.769	7.496	11	-0.761
**ESC**	GaFIE	Ga13G1827	Chr13:108,140,020–108,143,539	1113	13	370	41.541	6.506	0	-0.14
	GrFIE	Gorai.013G163900	Chr13:44,496,405–44,500,407	1179	13	392	44.23	7.496	5.5	-0.127
	GhFIE-A	Gh_A13G1198	A13:65,032,252–65,035,862	1182	13	393	44.289	7.268	4.5	-0.148
	GhFIE-D	Gh_D13G1494	D13:46,561,859–46,565,374	1113	13	370	41.542	6.105	-2	-0.139
**Su(z)12**	GaEMF2-1	Ga01G2053	Chr01:98,229,921–98,239,864	1887	20	628	71.463	8.109	13	-0.258
	GaEMF2-2	Ga07G0529	Chr07:5,640,221–5,647,886	1863	20	620	70.049	7.863	11	-0.3
	GrEMF2-1	Gorai.003G112000	Chr03:34,291,582–34,303,080	1890	21	629	71.44	7.898	12	-0.255
	GrEMF2-2	Gorai.001G051500	Chr01:4,887,828–4,896,308	1872	21	623	70.182	8.006	12.5	-0.295
	GhEMF2-1A^**b**^	Gh_A03G0526	A03:12,660,373–12,670,320	1887	20	628	71.554	8.240	14	-0.285
	GhEMF2-1D	Gh_D03G1003	D03:34,295,512–34,305,048	1887	20	628	71.343	8.111	13.5	-0.273
	GhEMF2-2A	Gh_A07G0381	A07:4,832,782–4,840,447	1872	20	623	70.279	7.738	10	-0.306
	GhEMF2-2D	Gh_D07G0444	D07:4,788,514–4,796,113	1872	20	623	70.276	7.891	11.5	-0.288
	GaVRN2	Ga01G2822	Chr01:112,751,961–112,760,094	1356	16	451	51.644	7.545	6.5	-0.417
	GrVRN2	Gorai.003G176300	Chr03:44,764,344–44,771,220	1281	15	426	48.763	7.013	3.5	-0.437
	GhVRN2-A	Gh_A03G0065	A03:1,028,419–1,037,603	1362	16	453	51.854	7.371	5.5	-0.414
	GhVRN2-D	Gh_D03G1592	D03:45,793,031–45,802,243	1362	16	453	51.789	7.545	6.5	-0.413
**p55**	GaMSI1	Ga13G0133	Chr13:1,403,099–1,405,249	1275	4	424	48.179	4.484	-26	-0.515
	GrMSI1	Gorai.013G014000	Chr13:960,697–963,463	1275	4	424	48.179	4.484	-26	-0.515
	GhMSI1-A	Gh_A13G0106	A13:1,257,122–1,259,271	1275	4	424	48.225	4.484	-26	-0.507
	GhMSI1-D	Gh_D13G0122	D13:1,231,942–1,234,124	1275	4	424	48.149	4.484	-26	-0.509

^a^Systematic designation of cotton PRC2 components was according to their *Arabidopsis* PRC2 orthologs and the chromosomal localization

^b^GhEMF2-1A, -1D, -2A, -2D were previouly reported as GhEMF2-A, -B, -C, -D, respectively [44, 45]

We also predicted the physiochemical properties of cotton PRC2 core components. The full-length coding sequences (CDS) of cotton PRC2 genes varied from 1113 to 2796 base pairs (bp), and consisted of 4 to 21 exons. The corresponding protein sequences ranged from 370 to 931 amino acid residues (aa) in length, with predicted molecular weights (MW) from 41.541 to 104.683 kDa, theoretical isoelectric points (pI) from 4.484 to 8.728, charges from -26 to 32, and grand average of hydropathy (GRAVY) value from -0.818 to -0.14 (Table 1).

### Phylogenetic and microsynteny analysis of cotton PRC2 core components

**Fig. 1:**
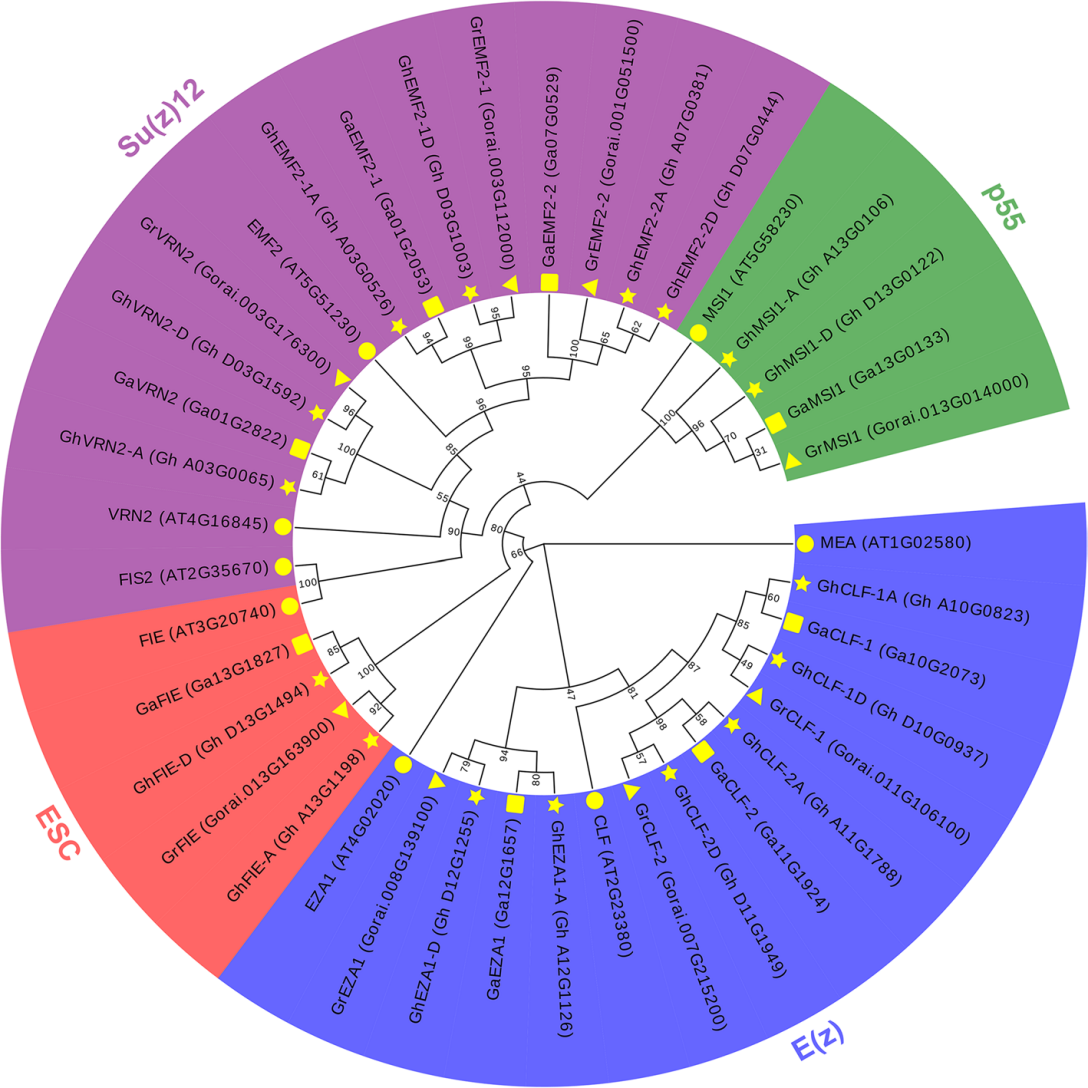
Phylogenetic analysis of PRC2 core components from three cotton species and *Arabidopsis*. The Neighbor-Joining phylogenetic tree was constructed by MEGA 7.0 using a bootstrap assessment of 1000 replicates. The blue, red, purple and green shaded regions indicate E(z), ESC, Su(z)12 and p55 subunits, respectively. The yellow circle, triangle, square and pentacle represent *Arabidopsis*, *G. raimondii*, *G. arboreum*, and *G. hirsutum*, respectively. The numbers at the branching nodes are the bootstrap values

To evaluate the evolutionary relationship of cotton PRC2 core components, a rootless phylogenetic tree was constructed based on the full-length protein sequences of cotton and *Arabidopsis* PRC2 proteins. Expectedly, cotton PRC2 proteins were classified into four groups as well as their *Arabidopsis* homologs. The E(z) group contained twelve CLF and EZA1/SWN homologs; the ESC group included four FIE equivalent; the Su(z)12 group was comprised of twelve EMF2 and VRN2-like proteins; and four MSI1 counterparts composed the p55 group. The cotton PRC2 components in each group tended to form closer clusters rather than with their *Arabidopsis* homologs. The GhPRC2 proteins encoded by Dt subgenome were grouped together with *G. raimondii* counterparts, while the At subgenome-derived PRC2 proteins were more closely related to *G. arboretum* homologs (Fig. 1), in consistent with cotton genome evolution [44].

**Fig. 2:**
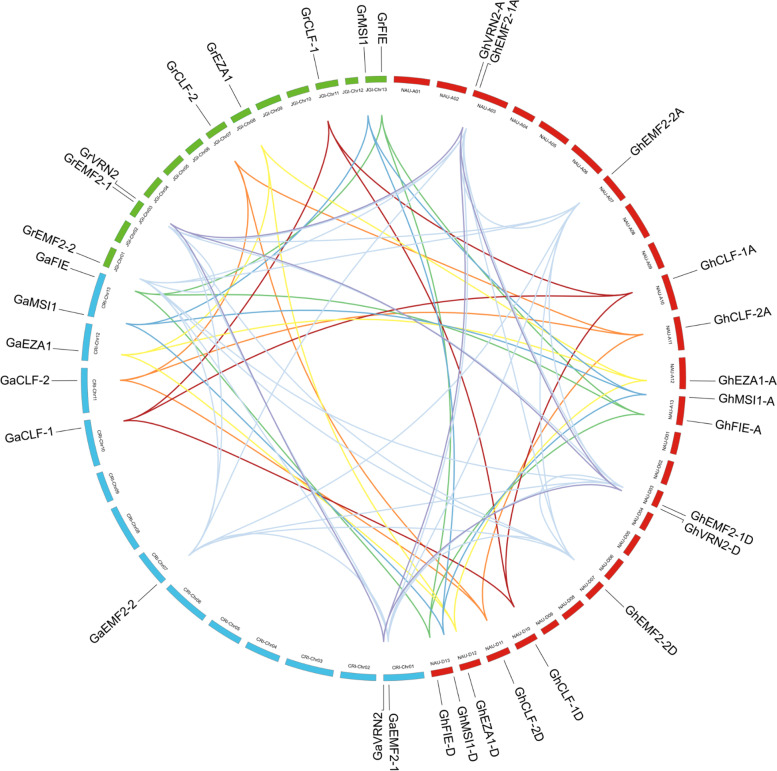
Chromosomal localization and microsynteny analysis of PRC2 genes from three cotton species. The chromosomal location and collinearity was evaluated by MCScanx and visualized with Circos. The green, blue and red boxes refer to chromosomes of *G. raimondii*, *G. arboreum*, and *G. hirsutum*, respectively. The chromosome numbers are marked inside the corresponding chromosome. The lines with different colors show the collinearity of cotton PRC2 genes

A microsynteny analysis based on the genomic DNA sequences of cotton PRC2 components was carried out to explore the chromosomal localization and evolutionary history. As shown in Fig. 2, cotton PRC2 genes were unevenly mapped on multiple chromosomes. Taken *G. hirsutum* as an example, chromosome A03, A13, D03 and D13 possessed two PRC2 genes each, whereas chromosome A07, A10, A11, A12, D07, D10, D11 and D12 contained only one PRC2 gene each. No PRC2 genes were found on the remaining chromosomes. GhPRC2 genes were preferentially localized near the terminus of these chromosomes in general. In addition, most of GhPRC2 genes derived from At- and Dt-subgenomes displayed a good collinearity with their homologs from A genome in *G. arboretum* and D genome in *G. raimondii*, respectively.

### Gene structure and protein domain architecture of GhPRC2 core components

**Fig. 3:**
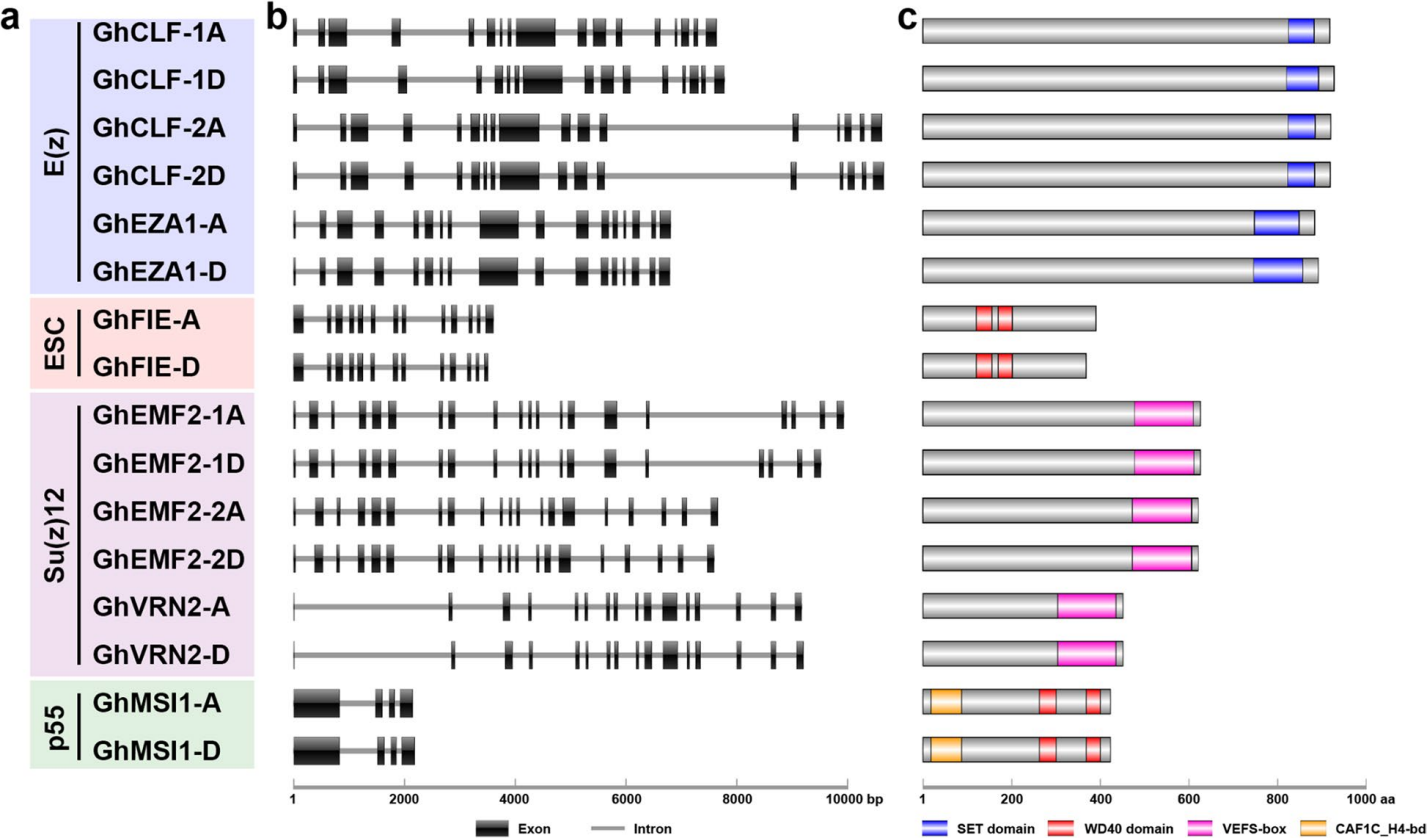
Gene and protein structures of *G. hirsutum* PRC2 core components. **a** The classification of *G. hirsutum* PRC2 core components. **b** Exon/intron distribution of *G. hirsutum* PRC2 genes. The black boxes and gray lines indicate exons and introns, respectively. **c** Conserved domain architecture of *G. hirsutum* PRC2 proteins according to the Pfam prediction. The blue, red, pink and orange boxes represent SET domain, WD40 domain, VEFS-box, and CAF1C_H4-bd domain, respectively

Considered the importance of upland cotton in textiles and oil industry, we focused our studies on GhPRC2 core components. We analyzed the exon–intron distributions to examine the gene structure of GhPRC2 genes. In spite of the variability of genomic DNA length, GhPRC2 genes in the same group shared the same number of exons and introns, which was distinct with other groups. For instance, six Su(z)12 members, *GhEMF2-1A*, *-1D*, *-2A*, *-2D*, and *GhVRN2-A*, *-D*, possessed 19 exons, with the maximum number of exons, whereas the p55 homologs, *GhMSI1-A* and *-D*, included only four exons. None of GhPRC2 genes was intronless (Fig. 3a and 3b).

The domain organization of a protein is usually closely related to its molecular function. To characterize the domain arrangement of GhPRC2 proteins, their full-length protein sequences were submitted to the Pfam and SMART servers. As shown in Fig. 3c, several conserved domains stood out (The detailed domain information was listed in Additional file 2: Table S2). The E(z) group proteins, GhCLF-1A, -1D, -2A, -2D, and GhEZA1-A, -D, carried a SET domain adjacent to the C terminus, which is an evolutionarily conserved, 130–160 aa-length sequence that is responsible for the lysine methyltransferases activity [8]. Two putative SANT (SWI3, ADA2, N-CoR and TFIIIB DNA-binding) domains, which may associate with DNA/histone binding and protein–protein interaction, were also present in these proteins predicted by the SMART server [50]. The ESC homologs, GhFIE1-A and -D contained two and four WD40 repeats predicted by Pfam and SMART, respectively. The Su(z)12 group members, GhEMF2-1A, -1D, -2A, -2D, and GhVRN2-A, -D, harbored an VEFS-box domain that may be involved in the interaction with E(z) proteins [51]. The p55-like proteins GhMSI1-A and -D possessed several WD40 repeats adjacent to the C terminus and a CAF1C_H4-bd domain near the N terminus, which could participate in the formation of chromatin assembly factor 1 (CAF-1) complex and the binding of histone H4 [52]. Generally, GhPRC2 proteins in the same group shared similar domain architecture, like their *Arabidopsis* counterparts [1].

### Subcellular localization of GhPRC2 core components

**Fig. 4:**
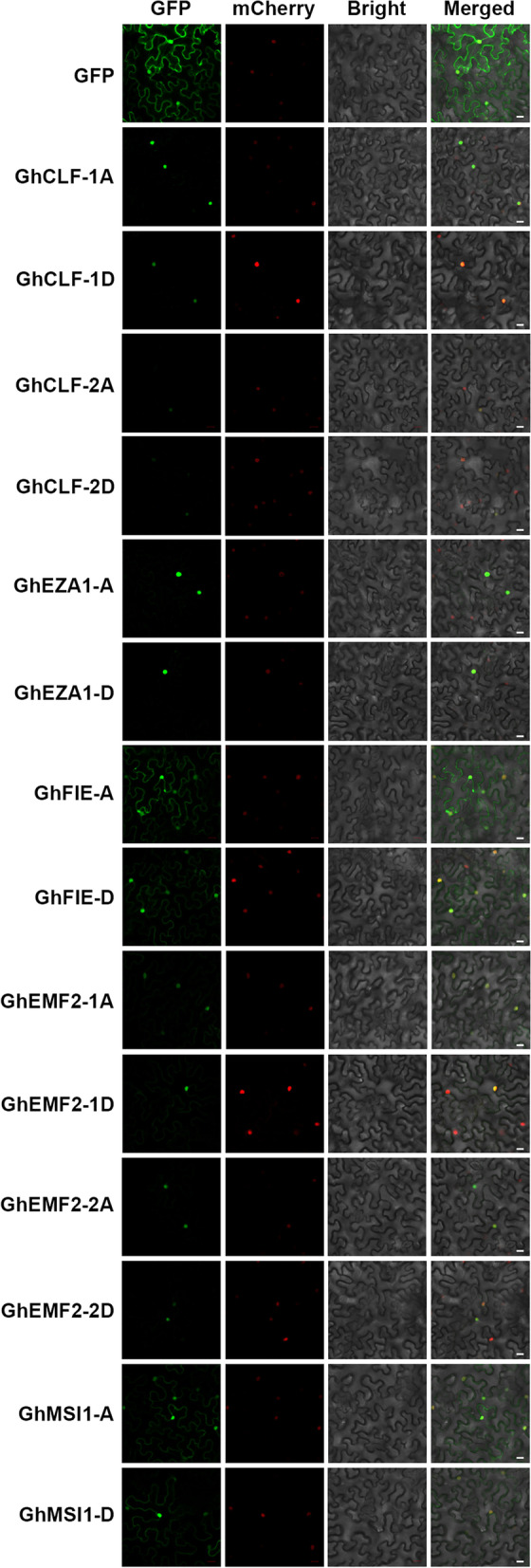
Subcellular localization of *G. hirsutum* PRC2 core components. The C-terminal GFP-fused *G. hirsutum* PRC2 recombinant constructs were transiently expressed in *N. benthamiana* leaves. And the subcellular localization of GhPRC2 proteins were determined by the GFP fluorescence signals. The empty vector pCAMBIA 1300:sGFP was used as a control. The *35S:*H2B-mCherry vector was used as a nuclear localization marker. The GFP and mCherry signals were collected with the confocal laser scanning microscopy and were shown in green and red, respectively. Scale bar = 20 μm

PRC2 plays a dominant role on depositing repressive H3K27me3 chromatic marks on the target loci, thus GhPRC2 components are predicted to be localized in the nucleus. To confirm their subcellular localization, the C-terminal Green Fluorescent Protein (GFP)-tagged GhPRC2 proteins driven by the CaMV 35S promoter were transiently expressed in tobacco leaves. As shown in Fig. 4, free GFP was strongly localized in both cytoplasm and nucleus, whereas the GFP fluorescent signals of most GhPRC2 fusion proteins were detected in the nucleus and colocalized with the nuclear localization signals, correlating with their potential regulatory functions on gene transcription. GhMSI1-A/D and GhFIE-A/D showed strong fluorescent signals in the nucleus and detectable fluorescent signals in the cytoplasm, in line with their *Arabidopsis* homologs [53]. However, it remains unclear whether GhFIE-A/D dynamically translocate from the cytoplasm to the nucleus driven by the direct phosphorylation by TOR kinase as *Arabidopsis* FIE [54]. Unfortunately, we have not successfully cloned the full-length CDS of GhVRN2-A/D.

### Protein–protein interactions of GhPRC2 core components

The four conserved subunits, E(z), Su(z)12, ESC, and p55 in *Drosophila* and their homologs in other species, usually form tetramer PRC2 complexes to achieve their molecular functions. To investigate the protein interactions of GhPRC2 core components, we constructed predicted protein interaction networks in the STRING database. The results showed that most GhPRC2 components interacted with at least one other GhPRC2 proteins. In particular, three E(z) group proteins (GhCLF-1A, -1D, and -2A) interacted with eight other GhPRC2 proteins. Interestingly, the interactions were not limited to the same subgenome (Additional file 3: Table S3 and Additional file 4: Figure S1). We also predicted potential interacting proteins of GhPRC2 components in the ccNET database. A bit differently, six Su(z)12 group components interact with the most number of the remaining GhPRC2 proteins, while E(z) group members interact with less number of GhPRC2 proteins than that in STRING database (Additional file 5: Table S4).

**Fig. 5:**
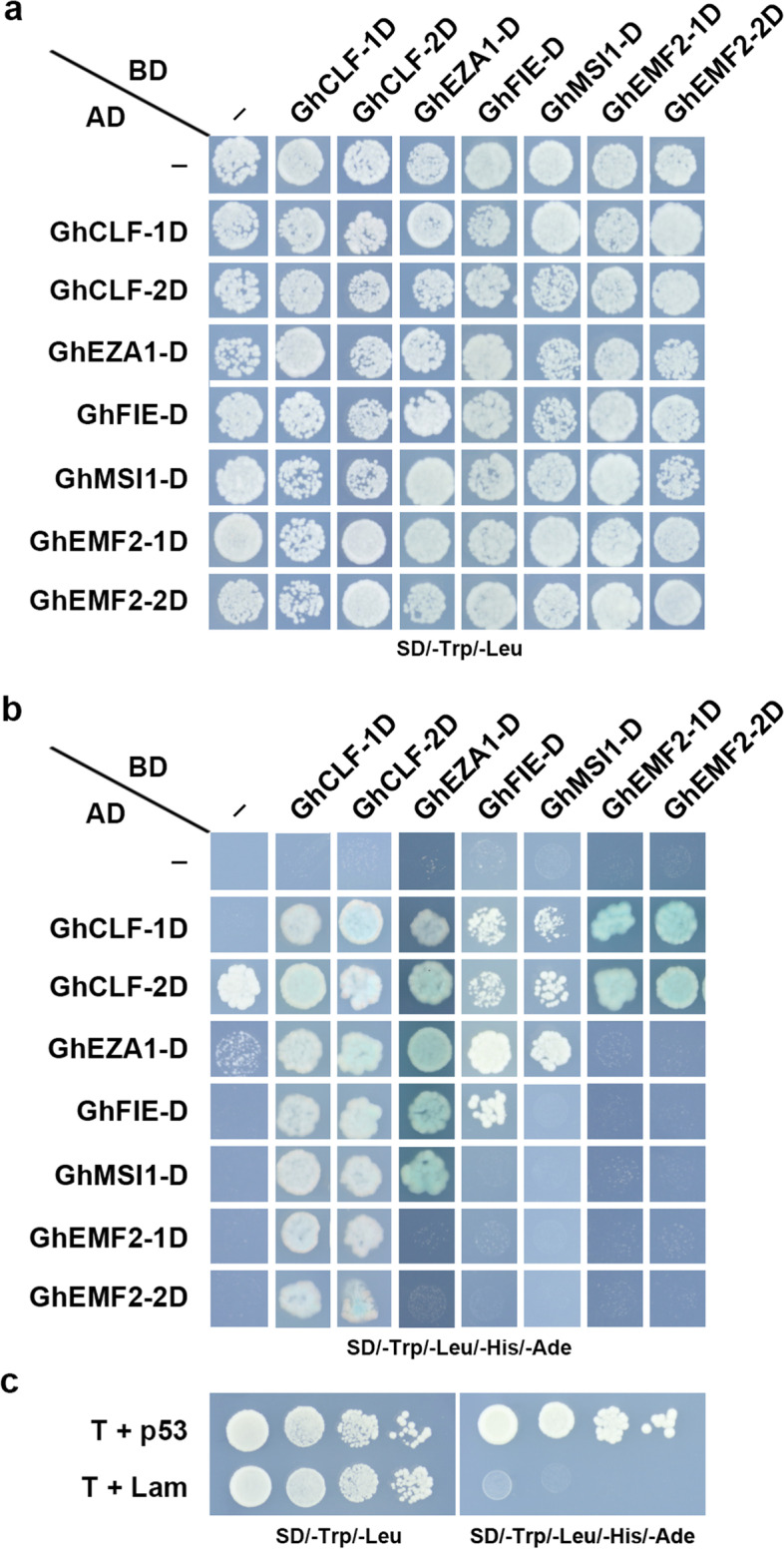
Protein interaction of *G. hirsutum* PRC2 core components in yeast two-hybrid assays. **a** and **b** Yeast cells co-transformed with empty AD (-)/AD fused *G. hirsutum* PRC2 proteins and BD/BD-fused PRC2 proteins were grown on SD/-Trp/-Leu and SD/-Trp/-Leu/-His/-Ade media in 10^–2^ dilution, respectively. **c** Yeast cells harboring pGBKT7-p53 (p53) and pGBKT7-Lam (Lam), co-transformed with pGADT7-T (T), and were used as positive and negative controls respectively, and grown on SD/-Trp/-Leu (left panels) and SD/-Trp/-Leu/-His/-Ade (right panels) media in 1, 10^–1^, 10^–2^, 10^–3^ dilutions (from left to right in each panel)

Furtherly, yeast two-hybrid assays were conducted to verify the potential protein interactions. Considering the high identity between At- and Dt-subgenomes derived GhPRC2 components, we investigated the one-by-one interactions of GhPRC2 proteins originated from Dt-subgenome. The results indicated that GhCLF-1D and GhCLF-2D interacted with all of GhPRC2 proteins; GhEZA1-D interacted with GhCLF-1D, GhCLF-2D, GhFIE-D, GhMSI1-D and itself; both GhFIE-D and GhMSI1-D could interact with GhCLF-1D, GhCLF-2D and GhEZA1-D; GhEMF2-1D and GhEMF2-2D interacted with GhCLF-1D and GhCLF-2D (Fig. 5). The results substantially agreed with the predicted protein interaction networks by the STRING database. In summary, these data suggested that GhPRC2 components may form multiple subunit complex. However, the physiological interactions are needed to be further validated *in planta*.

### Expression patterns of GhPRC2 genes in different tissues and development stages

**Fig. 6:**
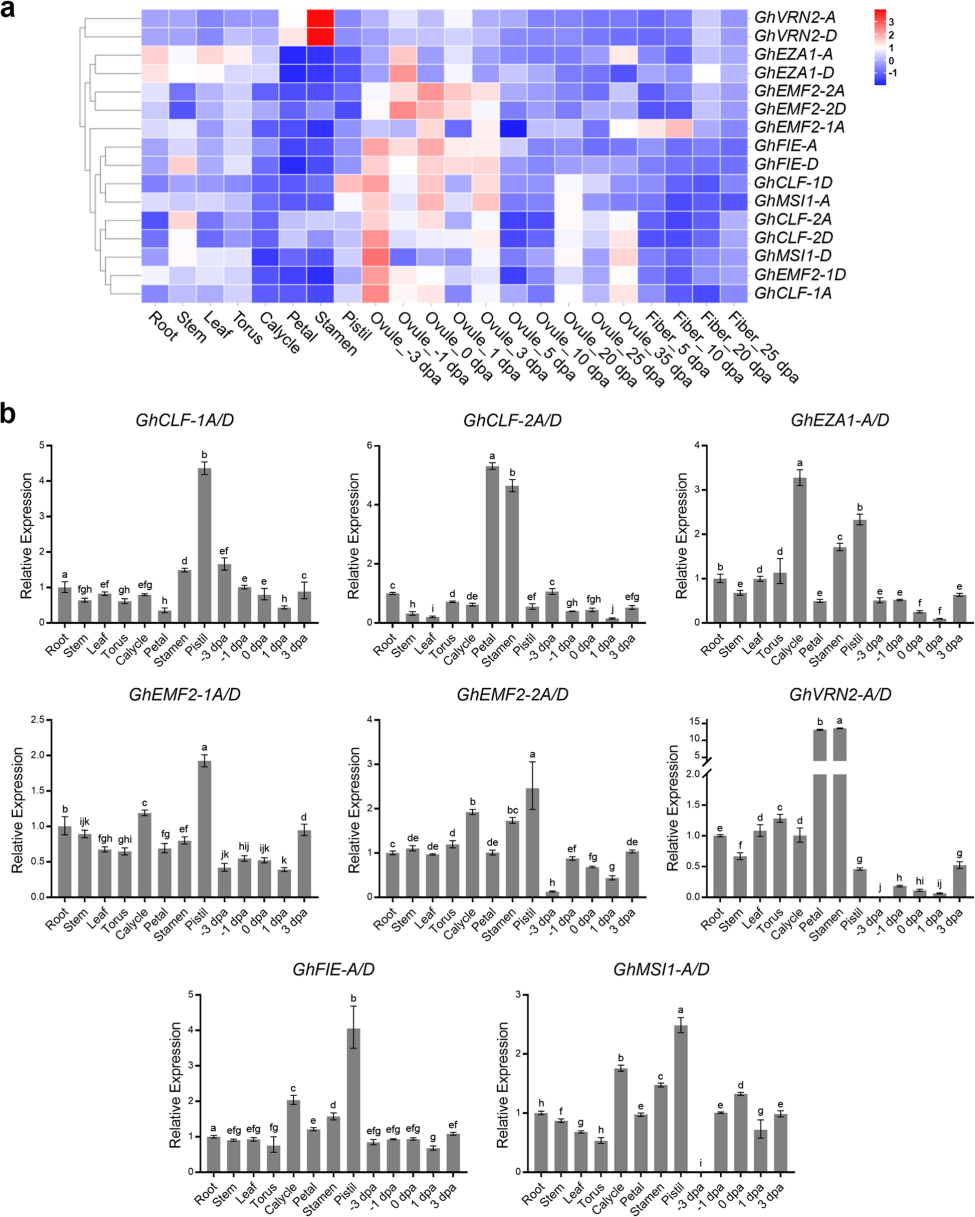
Tissue-specific expression patterns of *G. hirsutum* PRC2 genes. **a** Transcriptome expressions of *G. hirsutum* PRC2 genes in different tissues and developmental stages. The colors in each cell were based on the *z*-score normalized FPKM values. **b** Relative expression levels of *G. hirsutum* PRC2 genes in root, stem, leaf, torus, calycle, petal, stamen, pistil and -3 ~ 3 dpa ovules in qRT-PCR assays. The expression levels of each gene in root was set to 1 after all samples were normalized to *GhUBQ7* reference gene. The data show the mean ± SD of three biological replicates. Different letters indicate significant differences among different tissues (*p* < 0.05, one-way ANOVA)

The expression pattern is always associated with the biological functions of particular genes. To investigate the tissue specific expression of GhPRC2 genes, we analyzed a previously reported transcriptome data. The data showed that GhPRC2 genes were ubiquitously expressed in diverse tissues and different developmental stages, and the homologs originated from At- and Dt-subgenomes displayed similar expression patterns. Among E(z) group genes, *GhEZA1-A/D* showed the highest expression levels in most detected samples, *GhCLF-1A/D* the lowest, and *GhCLF2-A/D* the moderate. *GhEZA1-A/D* were relative lowly expressed in petal, stamen and pistil, but highly expressed in other tissues; *GhCLF-1A/D* displayed a low expression in calycle, petal, stamen, 10 and 20 dpa fiber, as well as a relative high expression in pistil, -3 ~ 3 dpa ovules; *GhCLF2-A/D* were also highly expressed in stem, besides with a similar global expression tend with *GhCLF-1A/D*. Likewise, *GhFIE-A/D* were highly expressed in stem and -3 ~ 3 dpa ovules. *GhVRN2-A/D* were highly expressed in all tissues with the highest expression level in petal and stamen. Four *GhEMF2* genes showed a generally common expression pattern with a high transcription level in -3 ~ 3 dpa ovules, however, the expression level of *GhEMF2-1A* and *GhEMF2-2A* was higher than their Dt-subgenome derived counterparts, respectively (Fig. 6a).

To valid the expression results, qRT-PCR assays were performed in different tissues from 2-month-old TM-1 plants in fully bloom. As shown in Fig. 6b, GhPRC2 genes showed similar but slightly varied expression in analyzed tissues. In line with the transcriptome data, most of GhPRC2 genes displayed relative high expressions in reproductive organs like calycle, petal, stamen and pistil. On the contrary, the high transcription levels were not detected in -3 ~ 3 dpa ovules. The relative expression levels of *GhEZA1-A/D* and *GhVRN2-A/D* were even less than half of that in roots. Besides, GhPRC2 genes could be clustered into several groups according to their expression profiles in qRT-PCR assays. For instance, *GhEMF2-1A/D*, *GhEMF2-2A/D*, *GhFIE-A/D* and *GhMSI1-A/D* shared generally common expression patterns distinct with the rest of GhPRC2 genes. Taken together, the high expressions of most GhPRC2 genes in reproductive organs implied that GhPRC2 components may be involved in the control of floral transition and the early stage of fiber development.

### Expression profiles of GhPRC2 genes under abiotic and biotic stresses

Cotton plants cultivated in the natural environment always suffer from diverse hostile stresses, including abiotic stresses like drought, salt, hot and cold, as well as biotic stresses like *Vd* infection. To explore the potential functions of GhPRC2 components in the adaption to abiotic stresses, we examined the expression profiles of GhPRC2 genes under different abiotic stresses based on the transcriptome data. The results indicated that the expressions of most GhPRC2 genes were significantly induced with varying degrees when exposed to drought. At 12 h after drought treatment, *GhCLF-1A/D* and *GhCLF-2A/D* remained a relative low expression, whereas *GhEZA1-A/D* and *GhMSI1-A/D* reached a high transcription level. When subjected to salt stress, most of GhPRC2 genes were rapidly up-regulated at 1 h, and then maintained relative stable expressions (*GhCLF-2D*, *GhEMF2-2A/D* and *GhVRN2-A/D*) or continuous increasements (*GhFIE-A/D* and the remaining genes). The opposite temperature stresses, hot and cold, resulted in complicated expression changes of *GhPRC2* genes. Following a continuous hot stress, the expressions of *GhCLF-1D* and *GhCLF-2A/D* firstly dramatically raised up and then declined; *GhFIE-A/D* expressions displayed an ongoing elevation; *GhEZA1-A/D* expressions were rapidly induced and kept a relative high levels; the rest genes were also slightly up-regulated at 1 h but with no obvious variation tend afterwards. Under longtime cold stress, most of GhPRC2 genes were induced at different timepoint. For example, the expression levels of *GhCLF-1A* and *GhVRN2-D* reached a maximum at 1 h, while *GhCLF-1D*, *GhCLF-2A/D*, *GhEMF2-1A/D* and *GhEMF2-2A/D* had the highest expression levels at 6 h (Additional file 6: Figure S2).

**Fig. 7:**
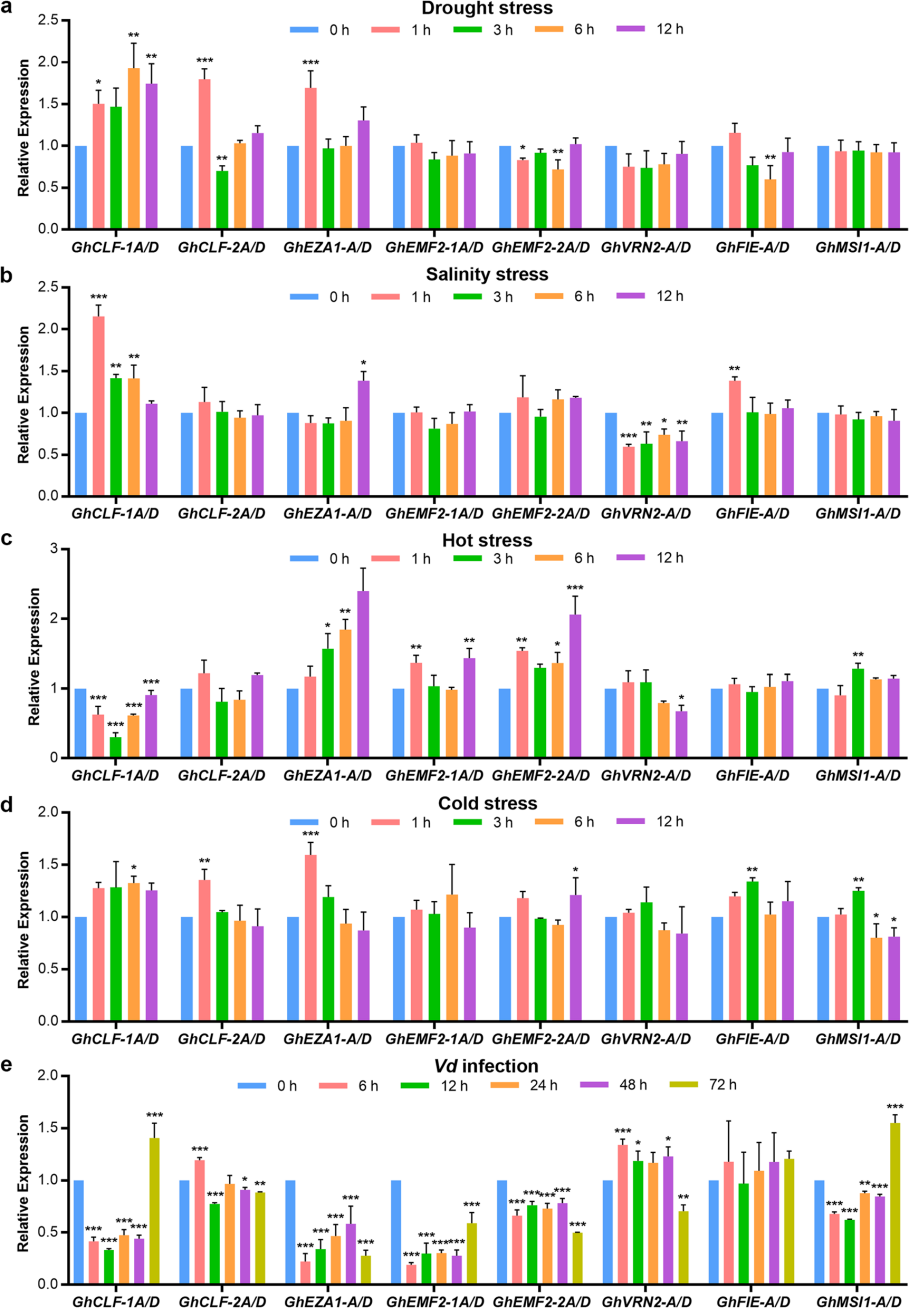
Expression profiles of *G. hirsutum* PRC2 genes in response to multiple abiotic stresses and *V. dahlia* infection. **a** to (**d**) Relative expression levels of *G. hirsutum* PRC2 genes at 0, 1, 3, 6, 12 h under drought (a), salinity (b), hot (c) and cold (d) stresses. **e** Relative expression levels of *G. hirsutum* PRC2 genes at 0, 6, 12, 24, 48, 72 h after *V. dahliae* infection. The expression levels of each gene at 0 h after each stress treatment was set to 1 after all samples were normalized to *GhUBQ7* reference gene. The data shown are the mean + SD of three biological replicates. Asterisks indicate significant differences with the expression level of corresponding genes at 0 h (*p < 0.05, **p < 0.01, ***p < 0.001, One-way ANOVA)

Furthermore, qRT-PCR assays were employed to verify the abiotic stresses-responsive expression of GhPRC2 genes. Unlike the transcriptome data, the relative expression levels of only a few GhPRC2 genes were changed under different abiotic stresses. *GhCLF-1A/D*, *GhCLF-2A/D* and *GhEZA1-A/D* displayed a rapidly drought-induced expressions at 1 h, while *GhEMF2-A/D* and *GhFIE-A/D* expressions were repressed at 6 h. Following the salinity stress, *GhCLF-1A/D* were significantly upregulated at 1 h and henceforth, whereas *GhVRN2-A/D* expressions were decreased. When exposed to excessive temperature, the expression of *GhEZA1-A/D*, *GhEMF2-1A/D* and *GhEMF2-2A/D* were raised up, however, *GhCLF-1A/D* expressions were declined. The cold treatment quickly elevated the transcriptions of *GhCLF-2A/D* and *GhEZA1-A/D* at 1 h, and relative slowly and slightly increased that of *GhCLF-1A/D*, *GhEMF2-2A/D*, *GhFIE-A/D*, and *GhMSI1-A/D* after 6 h. (Fig. 7a-d). Summarized the transcriptome and qRT-PCR results, the expressions of several GhPRC2 genes, such as *GhEZA1-A/D*, *GhCLF-1A/D* and *GhCLF-2A/D*, were responsive to diverse abiotic stresses, suggesting a potential regulatory role of GhPRC2 components on the tolerance to multiple environmental stimuli.

*Verticillium* wilt caused by soil-borne fungal pathogens *V. dahlia* or *V. albo-atrum* is one of the most destructive cotton diseases that leads to enormous yield and economic losses [55]. To investigate the possible roles of GhPRC2 components in cotton resistance to *Vd* infection, qRT-PCR assays were performed in roots from TM-1 seedlings inoculated with *Vd*. As shown in Fig. 7e, GhPRC2 genes showed distinct expression patterns within 0 ~ 72 h after *Vd* infection. The expressions of *GhCLF-1A/D* and *GhEMF2-1A/D* were dramatically decreased at 6 h and maintained relative low levels until 72 h. The transcriptions of *GhEZA1-A/D*, *GhEMF2-2A/D* and *GhMSI1-A/D* were also remarkably down-regulated at 6 h but slowly upswung later. The results suggested that the repression of these genes may be required for the response to *Verticillium* wilt. In contrast, the expressions of *GhCLF-2A/D* and *GhVRN2-A/D* were elevated at 6 h and kept a mild higher levels henceforth. The effect on *GhFIE-A/D* transcriptions was almost negligible.

## Discussion

### Identification of plant PRC2 core components

Up to now, PRC2 core components have been identified in various eukaryote species. The originally identified *Drosophila* PRC2 contains four core subunits encoded by single genes, E(z), Su(z)12, ESC, and Nurf55/p55 [1, 2]. In the unicellular green alga *O. lucimarinus*, there are one copy of E(z), Su(z)12, ESC equivalents each, and two p55 proteins [20]. By contrast, the compositions of PRC2 complexes display higher complicacy and diversification in higher plants. One striking feature is that PRC2 core subunits have a few homologs encoded by multi-gene families. The *Arabidopsis* genome encodes three E(z) homologs MEA, CLF and SWN, three Su(z)12 equivalents FIS2, EMF2, and VRN2, only one ESC counterpart FIE, and five p55 proteins MSI1-MSI5 [1–3, 15, 16]. Of note, the orthologs of *Arabidopsis* MEA and FIS2, two key modulators for endosperm and seed development, and that of VRN2, an important component of the flowering regulatory complex, have not been found in most higher plants. For example, two E(z) homologs OsCLF and OsiEZ1/OsSET1, two Su(z)12 homologs OsEMF2a and OsEMF2b, two ESC homolog OsFIE1 and OsFIE2, and one p55 protein OsRBAP3 have been identified to comprise the PRC2 complex in rice [17]. Likewise, maize PRC2 contains seven core components including one more E(z) protein [18, 19]; barley PRC2 consists of at least one E(z)-like protein, three Su(z)12 homologs, and one ESC counterpart [22]; the hexaploidy bread wheat genome encodes nine E(z) homologs, eight Su(z)12 homologs, six ESC homologs and six p55 proteins [23]. These cereal E(z) homologs are orthologs of *Arabidopsis* CLF and SWN, and the Su(z)12 homologs fall into the EMF2 clade [49]. A recent study in *M. truncatula* identified 31 PRC2 core components, containing two MEA ortholog and one VRN2 ortholog [24], which is distinct from those in cereals.

In the present study, we identified eight PRC2 core components in diploid *G. arboretum* and *G. raimondii*, including three E(z) homologs, three Su(z)12 equivalents, one ESC member and one p55-like proteins each, while the tetraploid *G. hirsutum* possesses 16 PRC2 proteins, twice as many as the diploid species (Table 1 and Fig. 1). The number of PRC2 core components in *G. raimondii* is consistent with that in a previous report, without regard to GrMSI2 [20]. The orthologs of *Arabidopsis* MEA and FIS2 are also absent in cotton species, in line with that in cereals [17–20, 23]. Nevertheless, the VRN2 orthologs have been identified, similar to *M. truncatula* [24]. Unsurprisingly, different cotton PRC2 members of the same clade display good collinearity, identical gene and protein structures, and similar subcellular localizations (Fig. 2–4). Taken together, PRC2 complexes are highly conserved during the evolution, because the four core components, E(z), Su(z)12, ESC and p55, can be identified in various species; however, their composition display a considerable variation among different species, which may due to the genome duplications and chromosome polyploidy.

### Protein interactions of plant PRC2 subunits

The four conserved PRC2 core subunits usually form functional hetero-tetramer complexes to introduce histone marker H3K27me3 on the target loci and to regulate the transcription [1–3]. In *Drosophila*, E(z) possesses the histone methyltransferase (HMTase) activity, the Su(z)12-p55 nucleosome-binding module anchors E(z) on the nucleosome, whereas ESC contributes to boost enzymatic activity. It is remarkable that *Drosophila* PRC2 show robust HMTase activity only as tetramer [56]. In plants, the increasement in the number of PRC2 core components leads to a more flexibility and complexity of PRC2 complexes. In *Arabidopsis*, at least three PRC2 complexes, FIS-PRC2, EMF2-PRC2, and VRN2-PRC2, play essential epigenetic regulatory roles during the life cycle [1–3, 15, 16]. In FIS-PRC2, FIE can interact with MEA and MSI1 but not with FIS2, whereas FIS2 can interact with MEA but not with other FIS proteins [57]. Similarly, the physical interactions have been detected between two E(z)-like proteins (SWN and CLF) and all three Su(z)12 members (FIS2, VRN2 and EMF2), E(z)-like proteins and the ESC homolog FIE, FIE and the p55 homolog MSI1, FIE and two Su(z)12 components (VRN2 and EMF2), MSI1 and VRN2/EMF2. However, no convincing evidence validates the interaction between CLF and SWN, although they are functional abundantly present in EMF–PRC2 and VRN–PRC2 [1]. In rice, two possible PRC2 complexes, OsFIE1-containing PRC2 (OsCLF/OsiEZ1, OsFIE1, OsEMF2a/2b, and OsRBAP3) and OsFIE2-containing (OsCLF/OsiEZ1, OsFIE2, OsEMF2a/2b, and OsRBAP3) PRC2, may have distinct roles in endosperm development, based on the genetic and molecular evidences [49]. Of note, two recent studies revealed that the imprinted gene *OsEMF2a* is essential for endosperm cellularization and genomic imprinting [58], and the mutation of *OsEMF2a* causes autonomous endosperm development and delayed cellularization[59], suggesting that OsEMF2a-containing PRC2 possesses a similar role as *Arabidopsis* FIS-PRC2 in rice endosperm development. A recent study in maize reported that both the E(z) homologs MEZ1/2/3 and the ESC homologs ZmFIE1/ZmFIE2 can strongly interact with all the remaining PRC2 components, while Su(z)12 homologs ZmEMF2-1/2–2 showed a relative weak interactions with other subunits. These results together with their expression patterns proposed that two PRC2 complexes, ZmFIE1-PRC2 (MEZ1/3, ZmFIE1, ZmEMF2-1 and ZmMSI1-1/1–2) and ZmFIE2-PRC2 (MEZ2/3, ZmFIE2, ZmEMF2-2 and ZmMSI1-1/1–2), may be entangled with the development of endosperm cells and other cell types, respectively [19].

We investigated the protein interactions of GhPRC2 core components in this study. Our Y2H results, together with the predicted protein interaction networks, indicated that most of GhPRC2 components interacted with at least one other GhPRC2 proteins. The E(z) homologs GhCLF-1D/-2D can interact with all the remaining PRC2 components, while GhEZA1-D interacts with other PRC2 members but not with the Su(z)12 homolog GhEMF2-1D/2D. The interactions between GhFIE-D and GhMSI1-D, GhFIE-D and GhEMF2-1D/2D were not detected (Fig. 5). It seems like that cotton E(z) group proteins not only contribute to the HMTase activity but also most likely provides the skeleton for the assembly of other PRC2 subunits, consisting with that in *Arabidopsis* and cereals [1, 19, 49, 57]. One possible reason for this discrepancy is that only the Dt-subgenome derived PRC2 components were detected, besides the divergences among different species. The interactions between PRC2 protein originated from different cotton subgenomes should be considered and furtherly validated in vivo.

### Expression patterns and potential biological roles of cotton PRC2 genes

The expression patterns of PRC2 components are critical for their biological functions. For instance, FIS-PRC2 plays essential roles on endosperm and seed development in *Arabidopsis* [1–3, 15, 16]. Disruption of *MEA*, an imprinted gene specifically expressed in the female gametophyte and the endosperm of developing seeds, leads to the generation of autonomous seeds without fertilization and parent-of-origin effects. Mutation of FIS2 causes similar defects in endosperm development [28]. In cereals like rice and maize, the functional divergence of PRC2 complexes is largely dependent on the differential expression of ECS homologs, as mentioned previously. *OsFIE1*, the only maternal-expressed imprinted PRC2 gene in rice endosperm, is specifically expressed in the endosperm, whereas *OsFIE2* and other PRC2 genes are expressed in a wide range of tissues [17]. Correlating with their differential expressions, several reports have revealed their overlapping and distinct roles in rice endosperm [49, 60, 61]. Likewise, *ZmFIE1* displays a maternal-specific expression pattern and is predominantly expressed in the endosperm, while *ZmFIE2* is expressed in a range of tissues [18, 62]; however, their substantial roles in maize development remains obscure.

The high throughput RNA sequencing enables us to investigate the expression profiles of particular genes/gene families and to predict their functions in species with large and complicated genomes. Using RNA-seq data, a study in bread wheat revealed that the PcG homologs within the A, B and D subgenomes show highly similar transcriptional profiles, whereas members in different clades display variable transcriptional activities [23]. Another work in *M. truncatula* explored various types of expression of PcG genes and predicted their functions in the regulation of development and response to various environmental stimuli [24]. A very recent study in rice indicated that PcG genes are differentially expressed in different tissues, and responded variably in different environmental stress [63].

In this study, we analyzed the transcript profiles of PRC2 genes in *G. hirsutum* though RNA-seq data and qRT-PCR assays. The tissue and developmental stage specific expression data indicate that: (i) GhPRC2 genes are ubiquitously expressed in various tissues and developmental stage, with high expression levels in reproductive organs; (ii) GhPRC2 homologs showed similar but slightly varied expression in analyzed tissues; (iii) GhPRC2 genes in different group display distinct expression patterns (Fig. 6). These results strongly suggested that GhPRC2 may be involved in cotton flowering and bolling. Indeed, *GhEMF2s* have been reported to repress the floral transition by modulating the expression of several floral regulators [47, 48]. Moreover, these results implied distinct roles of GhPRC2 components in different groups as well as GhPRC2 paralogs in the same group. For example, *GhEZA1-A/D* showed considerable expression levels in vegetative organs like roots, stems and leaves, while *GhCLF-1A/D* and *-2A/D* were highly expressed in reproductive organs like stamens and pistils. Our stress-responsive expression results indicate that the expression of several PRC2 genes are altered by multiple stresses. In particular, the transcription profiles of E(z) group genes, *GhEZA1-A/D*, *GhCLF-1A/D* and *GhCLF-2A/D*, can be responsive to almost all stress treatments including drought, salinity, hot, cold and *Vd* infection (Fig. 7), suggesting these components may play potential regulatory roles in the tolerance to various environmental stimuli. Interestingly, even exposed to single stress, GhPRC2 genes display remarkable differences in the trends and ranges of expression changes, in accordance with that in bread wheat and rice. It is noteworthy that the expression profiles of GhPRC2 genes from transcriptomic data and qRT-PCR results are not completely consistent. A possible explanation is that the former is based on the average fragments per kilobase of exon per million mapped fragments (FPKM) of two biological repeats, while the later is according to the average relative fold changes to the expression of the reference gene *GhUBQ7* of at least three biological replicates.

## Conclusion

The genome-wide identification and characterization of PRC2 core components in *G. hirsutum* provides important and extensive information on cotton PRC2 complexes, which will help to understand their molecular mechanisms and potential biological roles. More detailed in vivo studies are required to reveal the protein interaction mechanisms, the types and compositions of cotton PRC2 complexes, the functional conservation and divergence of cotton PRC2 core subunits in certain biological processes as well as in different cotton species.

## Methods

### Plant materials and growth conditions

The upland cotton *Gossypium hirsutum* L. acc. TM-1 and the tobacco *Nicotiana benthamiana* were used in this study. The TM-1 seeds originally obtained from the Institute of Cotton Research, Chinese Academy of Agricultural Sciences (CAAS), Anyang, China, were sterilized with 1% sodium hypochlorite, and germinated in a sterile dish covered with moist filter paper at 25 °C for 3 days. And then the uniform seedlings were transplanted in a phytotron at 25 °C under a light intensity of 100 μmol m^−2^ s^−1^ and a photoperiod of 16 h light/8 h dark, or in experimental plots under standard farming conditions at Henan University in Kaifeng, China. The *N. benthamiana* seeds stored in our lab were grown in the same phytotron.

For the cloning of cotton PRC2 genes, 3-week-old seedlings grown in the phytotron and about 2-month-old cotton plants in fully bloom grown in the experimental plots were harvested. For the tissue and organ specific expression assays, the indicated tissues were collected from TM-1 plants in fully bloom grown in the experimental plots. For the abiotic stress treatment, about 3-week-old seedlings in the two-leaf stage grown in the phytotron were exposed to different abiotic stresses, and the leaves were collected at 0, 1, 3, 6, 12 h after treatment. For drought and salinity stresses, the well-growth cotton seedlings were watered thoroughly with 20% (v/v) PEG-6000 and 200 mM NaCl solutions, respectively. For hot and cold stresses, the seedlings were transferred into a phytoincubator at 40 °C and 4 °C, respectively. For *Vd* infection, the seedlings were watered thoroughly with the spore suspension of *Vd* 991 (1 × 10^7^ spores/mL) to ensure they were successfully inoculated, and then the roots were collected at 0, 6, 12, 24, 48, and 72 h after inoculation. More than 10 plants were treated in each replicates, and at least three biological replicates were performed.

### Identification of PRC2 core components

The genomic data of *G. arboreum* (A2, CRI assembly), *G. raimondii* (D5, JGI assembly), and *G. hirsutum* (AD1, NAU assembly) were downloaded from the CottonFGD database (http://www.cottonfgd.org/) [64]. The protein sequences of *Arabidopsis* PRC2 core components were obtained from the TAIR database (http://www.arabidopsis.org/). A BLASTP search (Parameters: *e*-value, 1e-10; matrix, BLOSUM62; gap-open, 11; gap-extend, 1; filter, F) using *Arabidopsis* PRC2 proteins as queries was employed against the selected cotton genomic database to obtain cotton PRC2 homologs. The physiochemical parameters of cotton PRC2 core components, including the exon and intron numbers, the amino acid residue numbers, predicted molecular weights, theoretical isoelectric points, charges, and grand average of hydropathy values, were analyzed in the CottonFGD database.

### Phylogenetic analysis

The full-length protein sequences of PRC2 core components from *G. arboreum*, *G. raimondii*, *G. hirsutum* and *Arabidopsis* were used for the phylogenetic analysis. The multi-sequence alignment was carried out by ClustalX2, and the phylogenetic tree was constructed using the Neighbor-Joining method in MEGA7.0 [65]. The reliability of internal tree branches was assessed by the bootstrap method with 1000 replicates. The original tree was beautified on the Evolview server (https://www.evolgenius.info//evolview/) [66].

### Chromosome location and collinearity analysis

The chromosome location information of PRC2 core components from *G. arboreum*, *G. raimondii*, and *G. hirsutum* was retrieved from the corresponding genome annotation files in the CottonFGD database. The collinearity of PRC2 genes among three cotton species was evaluated by the MCScanx software (http://chibba.pgml.uga.edu/mcscan2/) [67]. The chromosomal location and collinearity was visualized with the Circos software [68].

### Gene structure and conserved protein domain analysis

The CDS sequences and the genome sequence of GhPRC2 core components were used to analyze the exon–intron distribution. The full-length GhPRC2 protein sequences were submitted to the Pfam (http://pfam.xfam.org/) [69] and SMART (http://smart.embl-heidelberg.de/) [70] servers to analyzed the conserved domains. Then the results were visualized with the IBS software [71].

### Subcellular localization assays

The coding sequences of GhPRC2 core components without the stop codons were PCR amplified from the upland cotton TM-1 seedling cDNA, and cloned into the pCAMBIA 1300:sGFP vector to generate the C-terminal GFP-fused constructs. The specific primers were listed in Additional file 7: Table S5. The recombinant plasmids and the control vector were transformed into *Agrobacterium tumefaciens* strain GV3101. The *Agrobacterium* and then co-infiltrated onto 3-week-old tobacco leaves with the GV3101 strain harboring the nuclear localization marker vector *35S:*H2B-mCherry. Two days after infiltration, the tobacco leaves were collected, observed and photographed with a Zeiss LSM 780 confocal laser scanning microscope according to the manufacturer’s manual (Zeiss, Germany).

### Prediction of protein–protein interactions

The predicted protein–protein interaction networks were generated with STRING (https://string-db.org) [72] using GhPRC2 protein sequences to search the *G. hirsutum* databases, and visualized with the Cytoscape software [73]. The detailed information was shown in Additional file 3: Table S3. We also predicted potential interacting proteins of GhPRC2 components in the ccNET database (http://structuralbiology.cau.edu.cn/gossypium), and the detailed information was listed in Additional file 5: Table S4.

### Yeast two-hybrid assays (Y2H)

The Y2H assays were performed according to the manufacturer’s instructions (Clontech, USA). The full-length coding sequences of GhPRC2 core components were cloned into the prey vector pGADT7 and the bait vector pGBKT7, in-frame with the GAL4 activation domain (AD) and DNA-binding domain (BD), respectively. The specific primers were listed in Additional file 7: Table S5. The recombinant prey plasmids and the bait plasmids were co-transformed into yeast strain AH109 and screened on the SD/-Trp/-Leu plates. The positive clones were cultured in the SD/-Trp/-Leu medium at 30 °C for 4–6 h. Then the yeast cultures were collected by centrifugation, resuspended in TE buffer (10 mM Tris–HCl, 1 mM EDTA, pH7.5) to 1.0 OD600, and screened on the SD/-Trp/-Leu and SD/-Trp/-Leu/-His/-Ade plates in 1, 10^–1^, 10^–2^, 10^–3^ dilutions after growing at 30 °C for 3 days. At least three biological replicates were performed.

### Transcriptomic expression analysis

The transcriptomic data of GhPRC2 core component genes in different tissues and under different abiotic stress conditions were retrieved from the CottonFGD and ccNET database. The average fragments per kilobase of exon per million mapped fragments (FPKM) of two biological repeats were calculated as the gene expression levels. Then the expression heatmaps were drawn according the *z*-score normalized FPKM values on the OmicShare platform (https://www.omicshare.com/tools).

### RNA isolation and quantitative real-time (qRT)-PCR

Total RNAs were isolated from the indicated plant tissues using the RNAprep Pure Plant Plus Kit (Polysaccharides & Polyphenolics-rich) (Tiangen, DP411, China). The first-strand cDNAs were synthesized from 1 ug total RNAs using the HiScript® III RT SuperMix Kit for qPCR (+ gDNA wiper) (Vazyme, R323-01, China). qRT-PCR was performed on the LightCycler® 480 system (Roche, Switzerland) using the ChamQ Universal SYBR qPCR Marster Mix Kit (Vazyme, Q711, China). *GhUBQ7* (GenBank accession No.DQ116441) was used as the internal references, and the relative expression levels of GhPRC2 genes were calculated using the 2^−ΔΔCT^ method. The gene specific primer sequences were designed on the qPCR Primer database (https://biodb.swu.edu.cn/qprimerdb/) [74], and listed in Additional file 7: Table S5. In each biological replicate, both the At and Dt-derived primer pairs were used. And at least three biological repeats were performed.

### Statistical analysis

The presented relative expression levels are expressed as mean ± SD of three biological replicates. Statistical analysis was assessed by one-way ANOVA.

## Supplementary Information

**Additional file 1: Table S1. **Identities between *Arabidopsis *and cotton PRC2 core components.

**Additional file 2: Table S2. **Predicted domain organization of *G. hirsutum* PRC2 proteins.

**Additional file 3: Table S3. **Predicted protein-protein interactions of *G. hirsutum* PRC2 core components in the STRING database.

**Additional file 4: Figure S1. **Predicted protein interaction networks of of *G. hirsutum* PRC2 core components.

**Additional file 5: Table S4. **Predicted interacting proteins of *G. hirsutum* PRC2 core components in the ccNET database.

**Additional file 6: Figure S2. **Transcriptome expressions of *G. hirsutum* PRC2 genes under diverse abiotic stresses.

**Additional file 7: Table S5. **Primers used in this study.

## Data Availability

All data generated or analyzed during this study are included in this article and its supplementary information files. Transcriptome data of GhPRC2 genes in Fig. 6A and Additional file 6: Figure S2 could be downloaded from the ccNET database with the gene ID.

## Abbreviations

PRC2: Polycomb Repressive Complex 2
H3K27me3: Tri-methylation at lysine 27 of histone H3
PcG: Polycomb group
PhoRC: Pho-repressive complex
PREs: Polycomb response elements
E(z): Enhancer of zeste
Su(z)12: Suppressor of zeste 12
ESC: Extra sex combs
Nurf55/p55: Nucleosome remodeling factor 55 kDa
*Vd*: *Verticillium dahlia*
SANT domain: SWI3, ADA2, N-CoR and TFIIIB DNA-binding domain
CaMV: The cauliflower mosaic virus
GFP: Green fluorescent protein
ABA: Abscisic acid
CDS: Coding sequences
qRT-PCR: Quantitative real‑time PCR
Y2H: Yeast two-hybrid assay
FPKM: Fragments per kilobase of exon per million mapped fragments
