# Supplementary material for: Genome-wide identification and characterization of polycomb repressive complex 2 core components in upland cotton (Gossypium hirsutum L.)
Source: BMC Plant Biol. 2023 Feb 1;23:66. doi: 10.1186/s12870-023-04075-4 (PMC9890721; doi:10.1186/s12870-023-04075-4)
Supplement: Supplementary file 1 — Additional file 1: Table S1. Identities between Arabidopsis and cotton PRC2 core components. [file 12870_2023_4075_MOESM1_ESM.docx]

**Table S1. Identities between *Arabidopsis* and cotton PRC2 core components.**

| **Arabidopsis PRC2 proteins** | **Cotton PRC2 proteins** | **Query coverage（%）** | **Total Score** | **Identities**  **(%)** | **E value** |
| --- | --- | --- | --- | --- | --- |
| **CLF**  **SWN/EZA1** | GaCLF-1  GaCLF-2  GrCLF-1  GrCLF-2  GhCLF-1A  GhCLF-1D  GhCLF-2A  GhCLF-2D  GaEZA1  GrEZA1 | 100  99  100  98  100  100  99  99  96  100 | 2822  2710  2787  2722  2832  2761  2707  2726  2422  2515 | 64.3  64.2  64  65  64.5  63.4  64.2  64.6  58  58.3 | 0  0  0  0  0  0  0  0  0  0 |
|  | GhEZA1-A  GhEZA1-D | 96  100 | 2527  2505 | 59.9  57.9 | 0  0 |
| **FIE** | GaFIE  GrFIE  GhFIE-A  GhFIE-D | 99  83  83  99 | 1587  1336  1584  1336 | 75.7  76  75.5  76 | 0  0  0  0 |
| **EMF2**  **VRN2**  **p55** | GaEMF2-1  GaEMF2-2  GrEMF2-1  GrEMF2-2  GhEMF2-1A  GhEMF2-1D  GhEMF2-2A  GhEMF2-2D | 99  99  99  99  99  99  99  99 | 2021  1936  2028  1976  2016  2043  1968  1959 | 64.4  63  64.7  64  64.5  64.7  63.6  63.5 | 0  0  0  0  0  0  0  0 |
|  | GaVRN2  GrVRN2 GhVRN2-A  GhVRN2-D | 89  89  89  89 | 1124  1089  1088  1089 | 54.4  54.2  54.4  54.2 | 2.70×10^-151^  1.17×10^-145^  1.22×10^-145^  8.01×10^-146^ |
|  | GaMSI1  GrMSI1  GhMSI1-A  GhMSI1-D | 100  100  100  100 | 2075  2075  2070  2075 | 91.5  91.5  91.3  91.5 | 0  0  0  0 |

BLASTp searches using the full-length Arabidopsis PRC2 protein sequences as queries were performed against the genomic data of *G. arboreum* (A2, CRI assembly), *G. raimondii* (D5, JGI assembly), and *G. hirsutum* (AD1, NAU assembly). In the case of multiple protein isoforms derived from the same gene, especially in *G. arboretum*, the data were generated from the isoform encoded by the principle transcript.
