# Supplementary material for: Genome-wide identification and characterization of polycomb repressive complex 2 core components in upland cotton (Gossypium hirsutum L.)
Source: BMC Plant Biol. 2023 Feb 1;23:66. doi: 10.1186/s12870-023-04075-4 (PMC9890721; doi:10.1186/s12870-023-04075-4)
Supplement: Supplementary file 2 — Additional file 2: Table S2. Predicted domain organization of G. hirsutum PRC2 proteins. [file 12870_2023_4075_MOESM2_ESM.docx]

**Table S2. Predicted domain organization of *G. hirsutum* PRC2 proteins.**

| **Subunit** | **Name** | **Domain Region** | **Domain Accession** | **Domain Description** | **E value** |
| --- | --- | --- | --- | --- | --- |
| 1. **Domain organization predicted in Pfam database** | | | | | |
| E(z)  ESC | GhCLF-1A  GhCLF-1D  GhCLF-2A  GhCLF-2D  GhEZA1-A  GhEZA1-D  GhFIE-A  GhFIE-D  GhEMF2-1A  GhEMF2-1D  GhEMF2-2A  GhEMF2-2D  GhVRN2-A  GhVRN2-D  GhMSI1-A  GhMSI1-D | 827-885  823-895  826-887  825-886  750-851  748-859  122-157  171-203  122-157  171-203 | PF00856  PF00856  PF00856  PF00856  PF00856  PF00856  PF00400  PF00400  PF00400  PF00400 | SET; SET domain  SET; SET domain  SET; SET domain  SET; SET domain  SET; SET domain  SET; SET domain  WD40; WD domain, G-beta repeat  WD40; WD domain, G-beta repeat  WD40; WD domain, G-beta repeat  WD40; WD domain, G-beta repeat | 2.20E-09  1.70E-09  1.50E-09  1.50E-09  3.60E-09  9.50E-09  0.0048  8.30E-05  0.0048  7.70E-05 |
| Su(z)12  p55 |  | 479-612  479-613  474-608  474-608  305-437  305-437  19-88  264-302  370-402  19-88  264-302  370-402 | PF09733  PF09733  PF09733  PF09733  PF09733  PF09733  PF12265  PF00400  PF00400  PF12265  PF00400  PF00400 | VEFS-Box; VEFS-Box of polycomb protein  VEFS-Box; VEFS-Box of polycomb protein  VEFS-Box; VEFS-Box of polycomb protein  VEFS-Box; VEFS-Box of polycomb protein  VEFS-Box; VEFS-Box of polycomb protein  VEFS-Box; VEFS-Box of polycomb protein  CAF1C_H4-bd; Histone-binding protein RBBP4 or subunit C of CAF1 complex  WD40; WD domain, G-beta repeat  WD40; WD domain, G-beta repeat  CAF1C_H4-bd; Histone-binding protein RBBP4 or subunit C of CAF1 complex  WD40; WD domain, G-beta repeat  WD40; WD domain, G-beta repeat | 3.70E-60  3.80E-61  4.60E-60  1.30E-60  1.40E-58  3.90E-59  1.50E-26  1.90E-06  0.19  1.50E-26  1.90E-06  0.19 |
| 1. **Domain organization predicted in SMART database** | | | | | |
| E(z)  ESC | GhCLF-1A  GhCLF-1D  GhCLF-2A  GhCLF-2D  GhEZA1-A  GhEZA1-D  GhFIE-A  GhFIE-D  GhEMF2-1A  GhEMF2-1D  GhEMF2-2A  GhEMF2-2D  GhVRN2-A  GhVRN2-D  GhMSI1-A  GhMSI1-D | 202-252  546-596  771-892  202-252  545-595  771-892  201-251  548-598  773-894  200-250  547-597  772-893  737-858  737-866  67-114  117-157  160-203  229-266 | SM00717  SM00717  SM00317  SM00717  SM00717  SM00317  SM00717  SM00717  SM00317  SM00717  SM00717  SM00317  SM00317  SM00317  SM00320  SM00320  SM00320  SM00320 | SANT SWI3, ADA2, N-CoR and TFIIIB DNA-binding domains  SANT SWI3, ADA2, N-CoR and TFIIIB DNA-binding domains  SET (Su(var)3-9, Enhancer-of-zeste, Trithorax) domain  SANT SWI3, ADA2, N-CoR and TFIIIB DNA-binding domains  SANT SWI3, ADA2, N-CoR and TFIIIB DNA-binding domains  SET (Su(var)3-9, Enhancer-of-zeste, Trithorax) domain  SANT SWI3, ADA2, N-CoR and TFIIIB DNA-binding domains  SANT SWI3, ADA2, N-CoR and TFIIIB DNA-binding domains  SET (Su(var)3-9, Enhancer-of-zeste, Trithorax) domain  SANT SWI3, ADA2, N-CoR and TFIIIB DNA-binding domains  SANT SWI3, ADA2, N-CoR and TFIIIB DNA-binding domains  SET (Su(var)3-9, Enhancer-of-zeste, Trithorax) domain  SET (Su(var)3-9, Enhancer-of-zeste, Trithorax) domain  SET (Su(var)3-9, Enhancer-of-zeste, Trithorax) domain  WD40 repeats  WD40 repeats  WD40 repeats  WD40 repeats | 1.9  2.4  2.20E-32  1.9  2.4  6.30E-31  33  0.12  2.30E-32  18  0.12  2.30E-32  4.40E-33  3.00E-32  140  9.60E-05  6.90E-08  94 |
| Su(z)12  p55 |  | 67-114  117-157  160-203  229-266  278-319  324-365  -  -  -  -  -  -  115-154  167-207  216-256  262-302  306-346  363-403  115-154  167-207  216-256  262-302  306-346  363-403 | SM00320  SM00320  SM00320  SM00320  SM00320  SM00320  -  -  -  -  -  -  SM00320  SM00320  SM00320  SM00320  SM00320  SM00320  SM00320  SM00320  SM00320  SM00320  SM00320  SM00320 | WD40 repeats  WD40 repeats  WD40 repeats  WD40 repeats  WD40 repeats  WD40 repeats  -  -  -  -  -  -  WD40 repeats  WD40 repeats  WD40 repeats  WD40 repeats  WD40 repeats  WD40 repeats  WD40 repeats  WD40 repeats  WD40 repeats  WD40 repeats  WD40 repeats  WD40 repeats | 140  0.00034  6.90E-08  94  71  0.035  -  -  -  -  -  -  21  0.0047  0.1  2.80E-08  0.00024  0.00087  21  0.0047  0.1  2.80E-08  0.00024  0.00087 |

-, not defined.
