# Supplementary figures and images for: Genome-wide identification and characterization of polycomb repressive complex 2 core components in upland cotton (Gossypium hirsutum L.)
Source: BMC Plant Biol. 2023 Feb 1;23:66. doi: 10.1186/s12870-023-04075-4 (PMC9890721; doi:10.1186/s12870-023-04075-4)

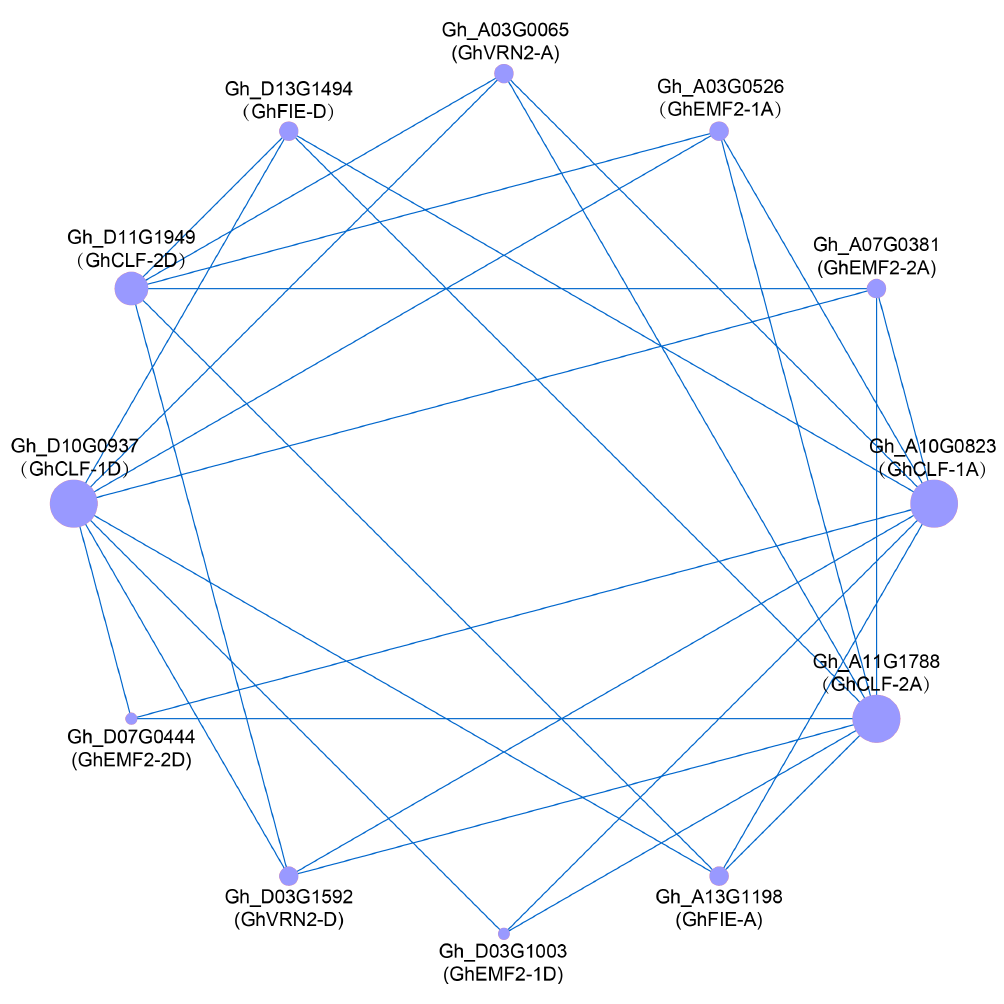

Supplement: Supplementary file 4 — Additional file 4: Figure S1. Predicted protein interaction networks of of G. hirsutum PRC2 core components. [file 12870_2023_4075_MOESM4_ESM.tiff]

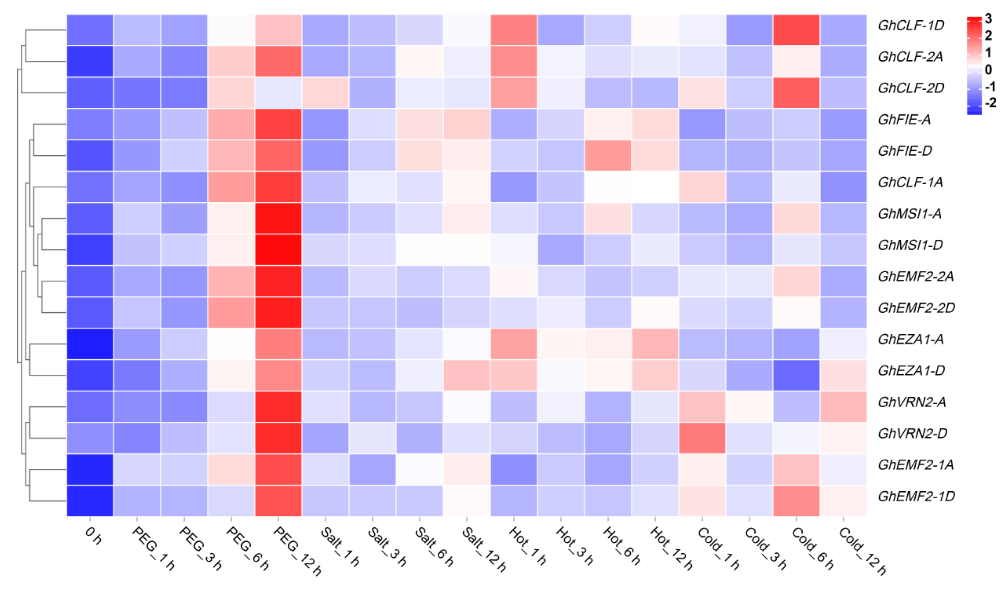

Supplement: Supplementary file 6 — Additional file 6: Figure S2. Transcriptome expressions of G. hirsutum PRC2 genes under diverse abiotic stresses. [file 12870_2023_4075_MOESM6_ESM.tiff]
