# Supplementary material for: Genome-wide identification and characterization of polycomb repressive complex 2 core components in upland cotton (Gossypium hirsutum L.)
Source: BMC Plant Biol. 2023 Feb 1;23:66. doi: 10.1186/s12870-023-04075-4 (PMC9890721; doi:10.1186/s12870-023-04075-4)
Supplement: Supplementary file 5 — Additional file 5: Table S4. Predicted interacting proteins of G. hirsutum PRC2 core components in the ccNET database. [file 12870_2023_4075_MOESM5_ESM.docx]

**Table S4. Predicted interacting proteins of *G. hirsutum* PRC2 core components in the ccNET database.**

| **Name** | **TOP10 predicted interacting proteins** |
| --- | --- |
| GhCLF-1A | Gh_D04G1524, Gh_A08G1183, Gh_D08G1467, Gh_D05G2141, Gh_A01G0023, Gh_A03G0526 (GhEMF2-1A), Gh_D01G0107, Gh_A10G1886, Gh_D10G2156, Gh_A03G1540 |
| GhCLF-1D | Gh_D07G0712, Gh_D01G0022, Gh_D10G2156, Gh_A03G1540,  Gh_D05G2540, Gh_A12G0964, Gh_A05G2280,  Gh_D12G1076, Gh_D13G1494 (GhFIE-D), Gh_A10G1886 |
| GhCLF-2A | Gh_D13G0122 (GhMSI1-D), Gh_A12G0964, Gh_A02G0245, Gh_A06G0273, Gh_A03G0065 (GhVRN2-A), Gh_D13G0518, Gh_D13G1494 (GhFIE-D), Gh_D12G1076, Gh_D02G1080, Gh_D03G1592 (GhVRN2-D) |
| GhCLF-2D | Gh_A07G0381 (GhEMF2-2A), Gh_D13G1494 (GhFIE-D), Gh_D07G0444 (GhEMF2-2D), Gh_A03G1926, Gh_D01G0107, Gh_A05G3068, Gh_A03G0065 (GhVRN2-A), Gh_Sca123729G01, Gh_A05G1920, Gh_D13G0518 |
| GhEZA1-A | Gh_A03G0065 (GhVRN2-A), Gh_D03G1422, Gh_A04G0980, Gh_A03G0163, Gh_A12G0772, Gh_A07G2179, Gh_D04G1524, Gh_D01G0107, Gh_A05G3068, Gh_D10G1454 |
| GhEZA1-D | Gh_D03G1003 (GhEMF2-1D), Gh_D02G2365, Gh_A12G0964, Gh_D03G1592 (GhVRN2-D), Gh_A04G0978, Gh_A13G0606, Gh_D03G1422, Gh_A03G1540, Gh_D01G0022, Gh_A13G1198 (GhFIE-A) |
| GhFIE-A | Gh_D04G1936, Gh_D12G1255 (GhEZA1-D), Gh_D04G1935, Gh_D13G0122 (GhMSI1-D), Gh_A05G3458,  Gh_D03G1003 (GhEMF2-1D), Gh_D10G0937 (GhCLF-1D), Gh_D05G2695, Gh_A07G0381 (GhEMF2-2A), Gh_A11G1788 (GhCLF-2A) |
| GhFIE-D | Gh_D11G1949 (GhCLF-2D), Gh_A05G3458, Gh_A11G1788 (GhCLF-2A), Gh_A01G0023, Gh_D10G0937 (GhCLF-1D), Gh_A12G1126 (GhEZA1-A), Gh_A05G2431, Gh_A07G0381 (GhEMF2-2A), Gh_D01G0022, Gh_D04G1935 |
| GhMSI1-A | Gh_D10G0399, Gh_A05G3068, Gh_D09G0612, Gh_D01G0135, Gh_A03G1490, Gh_D05G2262, Gh_A08G0294, Gh_D10G1731, Gh_A05G3458, Gh_D05G2930 |
| GhMSI1-D | Gh_A11G1788 (GhCLF-2A), Gh_A08G0045, Gh_D09G0169, Gh_D06G1504, Gh_A08G1969, Gh_D07G0227, Gh_D07G0226, Gh_D06G0508, Gh_D08G2361, Gh_D01G0135 |
| GhEMF2-1A | Gh_A10G0823 (GhCLF-1A), Gh_A05G0149, Gh_D05G0213, Gh_D12G1255 (GhEZA1-D), Gh_A12G1126 (GhEZA1-A),  Gh_A11G1788 (GhCLF-2A), Gh_D13G1494 (GhFIE-D), Gh_D11G1949 (GhCLF-2D), Gh_A13G1198 (GhFIE-A), Gh_D10G0937 (GhCLF-1D) |
| GhEMF2-1D | Gh_D12G1255 (GhEZA1-D), Gh_A10G0823 (GhCLF-1A), Gh_A13G1198 (GhFIE-A), Gh_D10G0937 (GhCLF-1D), Gh_D11G1949 (GhCLF-2D), Gh_A12G1126 (GhEZA1-A), Gh_A05G0149, Gh_D05G0213, Gh_A11G1788 (GhCLF-2A), Gh_D13G1494 (GhFIE-D) |
| GhEMF2-2A | Gh_D11G1949 (GhCLF-2D), Gh_A12G1126 (GhEZA1-A), Gh_D05G0213, Gh_D13G1494 (GhFIE-D), Gh_A13G1198 (GhFIE-A), Gh_A11G1788 (GhCLF-2A), Gh_A10G0823 (GhCLF-1A), Gh_A05G0149, Gh_D12G1255 (GhEZA1-D), |
|  | Gh_D10G0937 (GhCLF-1D) |
| GhEMF2-2D | Gh_D11G1949 (GhCLF-2D), Gh_A05G0149, Gh_D12G1255 (GhEZA1-D), Gh_A12G1126 (GhEZA1-A), Gh_A10G0823 (GhCLF-1A), Gh_D05G0213, Gh_A11G1788 (GhCLF-2A), Gh_D10G0937 (GhCLF-1D), Gh_D13G1494 (GhFIE-D), Gh_A13G1198 (GhFIE-A) |
| GhVRN2-A | Gh_A12G1126 (GhEZA1-A), Gh_A11G1788 (GhCLF-2A), Gh_D11G1949 (GhCLF-2D), Gh_D12G1255 (GhEZA1-D), Gh_A10G0823 (GhCLF-1A), Gh_D10G0937 (GhCLF-1D), Gh_A13G1198 (GhFIE-A), Gh_A05G0149, Gh_D13G1494 (GhFIE-D), Gh_D05G0213 |
| GhVRN2-D | Gh_D12G1255 (GhEZA1-D), Gh_D11G1949 (GhCLF-2D), Gh_A11G1788 (GhCLF-2A), Gh_A05G0149, Gh_D10G0937 (GhCLF-1D), Gh_A10G0823 (GhCLF-1A), Gh_A12G1126 (GhEZA1-A), Gh_D13G1494 (GhFIE-D), Gh_D05G0213, Gh_A13G1198 (GhFIE-A) |
