# Supplementary material for: Genome-wide identification and characterization of polycomb repressive complex 2 core components in upland cotton (Gossypium hirsutum L.)
Source: BMC Plant Biol. 2023 Feb 1;23:66. doi: 10.1186/s12870-023-04075-4 (PMC9890721; doi:10.1186/s12870-023-04075-4)
Supplement: Supplementary file 7 — Additional file 7: Table S5. Primers used in this study. [file 12870_2023_4075_MOESM7_ESM.docx]

**Table S5. Primers used in this study.**

| **Primer name** | **Sequence (5’-3’)** |
| --- | --- |
| **Primers for cloning and subcellular localization** | |
| *35S:GhCLF-1A/D-*F | CTTGACGAGCTCGGTACC-ATGACAGCGAAACCTTCGCGT |
| *35S:GhCLF-1A/D-*R | CACCATGTCGACTCTAGA-AGCAAGTTTCTTAGCTCGT |
| *35S:GhCLF-2A/D-*F | CTTGACGAGCTCGGTACC-ATGGCGGCGAAATCTTCGCCCT |
| *35S:GhCLF-2A/D-*R | CACCATGTCGACTCTAGA-AGCAAGCTTCTTGGCACGT |
| *35S:GhEZA1-AD-*F | CTTGACGAGCTCGGTACC-ATGGTCTCTAAAGGGAGCGAC |
| *35S:GhEZA1-AD-*R | CACCATGTCGACTCTAGA-ATGAGACTGGTGTTTCTTC |
| *35S:GhFIE-A-*F | CTTGACGAGCTCGGTACC-ATGGCCAAGACGCCGATAGG |
| *35S:GhFIE-A-*R | CACCATGTCGACTCTAGA-ACGGCAGCATTGCACAAC |
| *35S:GhFIE-D-*F | CTTGACGAGCTCGGTACC-ATGGCAAAGACGCCGATAGG |
| *35S:GhFIE-D-*R | CACCATGTCGACTCTAGA-GTTGGTCTGGACAGAATC |
| *35S:GhEMF2-12A/D-*F | CTTGACGAGCTCGGTACC-ATGCCGGGCATTCCTTTAGTG |
| *35S:GhEMF2-1A/D-*R | CACCATGTCGACTCTAGA-GCCTTTTATAGGATGCAA |
| *35S:GhEMF2-2A-R* | CACCATGTCGACTCTAGA-GCCTTTTATCGAATCCAA |
| *35S:GhEMF2-2D-R* | CACCATGTCGACTCTAGA-GCCTTTTATCAAATCCGA |
| *35S:GhMSI1-AD-*F | CTTGACGAGCTCGGTACC-ATGGGGAAAGACGAGGAGGAG |
| *35S:GhMSI1-A-*R | CACCATGTCGACTCTAGA-AGAACCTTTGGCAAATTC |
| *35S:GhMSI1-D-*R | CACCATGTCGACTCTAGA-AGAGCCTTTGGCAGATTC |
| **Primers for yeast two-hybrid assays** | |

| AD-*GhCLF-1D*-F | GATTACGCTCATATG-ATGACAGCGAAACCTTCGCGT |
| --- | --- |
| AD-*GhCLF-1D*-R | ACCCGGGTGGAATTC-TTAAGCAAGTTTCTTAGCTCGT |
| AD-*GhCLF-2D*-F | GATTACGCTCATATG-ATGGCGGCGAAATCTTCGCCCT |
| AD-*GhCLF-2D*-R | ACCCGGGTGGAATTC-TTAAGCAAGCTTCTTGGCACGT |
| AD-*GhEZA1-D*-F | GATTACGCTCATATG-ATGGTCTCTAAAGGGAGCGA |
| AD-*GhEZA1-D*-R | ACCCGGGTGGAATTC-TTAATGAGACTGGTGTTTCTT |
| AD-*GhFIE-D*-F | GATTACGCTCATATG-ATGGCAAAGACGCCGATAGGGCT |
| AD-*GhFIE-D*-R | ACCCGGGTGGAATTC-TCAGTTGGTCTGGACAGAAT |
| AD-*GhMSI1-D*-F | GATTACGCTCATATG-ATGGGGAAAGACGAGGAGGA |
| AD-*GhMSI1-D*-R | ACCCGGGTGGAATTC-TTAAGAGCCTTTGGCAGATT |
| AD-*GhEMF2-12D*-F | GATTACGCTCATATG-ATGCCGGGCATTCCTTTAGT |
| AD-*GhEMF2-1D*-R | ACCCGGGTGGAATTC-TTAGCCTTTTATAGGATGCA |
| AD-*GhEMF2-2D*-R | ACCCGGGTGGAATTC-TTAGCCTTTTATCAAATCCGA |
| AD-*GhVRN2-D*-F | GATTACGCTCATATG-ATGGCTGCTTTAAAACCGCCA |
| AD-*GhVRN2-D*-R | ACCCGGGTGGAATTC-TCAGCTTTTCATAGCATCCGA |
| BD-*GhCLF-1D*-F | CATATGGCCATGGAG-ATGACAGCGAAACCTTCGCGT |
| BD-*GhCLF-1D*-R | GGATCCCCGGGAATTC -TTAAGCAAGTTTCTTAGCTCGT |
| BD-*GhCLF-2D*-F | CATATGGCCATGGAG-ATGGCGGCGAAATCTTCGCCCT |
| BD-*GhCLF-2D*-R | GGATCCCCGGGAATTC -TTAAGCAAGCTTCTTGGCACGT |
| BD-*GhEZA1-D*-F | CATATGGCCATGGAG-ATGGTCTCTAAAGGGAGCGA |
| BD-*GhEZA1-D*-R | GGATCCCCGGGAATTC-TTAATGAGACTGGTGTTTCTT |
| BD-*GhFIE-D*-F | CATATGGCCATGGAG-ATGGCAAAGACGCCGATAGGGCT |
| BD-*GhFIE-D*-R | GGATCCCCGGGAATTC-TCAGTTGGTCTGGACAGAAT |
| BD-*GhMSI1-D*-F | CATATGGCCATGGAG-ATGGGGAAAGACGAGGAGGA |
| BD-*GhMSI1-D*-R | GGATCCCCGGGAATTC-TTAAGAGCCTTTGGCAGATT |
| BD-*GhEMF2-12D*-F | CATATGGCCATGGAG-ATGCCGGGCATTCCTTTAGT |
| BD-*GhEMF2-1D*-R | GGATCCCCGGGAATTC-TTAGCCTTTTATAGGATGCA |
| BD-*GhEMF2-2D*-R | GGATCCCCGGGAATTC-TTAGCCTTTTATCAAATCCGA |
| BD-*GhVRN2-D*-F | CATATGGCCATGGAG-ATGGCTGCTTTAAAACCGCCA |
| BD-*GhVRN2-D*-R | GGATCCCCGGGAATTC-TCAGCTTTTCATAGCATCCGA |

| **Primers for qRT-PCR*** | |
| --- | --- |
| *GhCLF-1A*-RT-F | TTGTGAGTGGAGGTGTTTCGCC |
| *GhCLF-1A*-RT-R | CCTTTTGCGAAGAAGACGTAGCA |
| *GhCLF-1D*-RT-F | CCAGTTAAGAATGCTGTTCGCCC |
| *GhCLF-1D*-RT-R | TCGCTGCAGATAAGTGCCTCAC |
| *GhCLF-2A*-RT-F  *GhCLF-2A*-RT-R  *GhCLF-2D*-RT-F  *GhCLF-2D*-RT-R  *GhEZA1-A*-RT-F  *GhEZA1-A*-RT-R  *GhEZA1-D*-RT-F  *GhEZA1-D*-RT-R  *GhFIE-A*-RT-F  *GhFIE-A*-RT-R | ACAGCTAACAGCTGACCGATCT  GCATGCCTAGTGCATCTTTTTGCC  ATGTGGCCTGCATTGCTATCGT  TGGGGAATAGGTGAGGCGTCTT  TGGTCTCTAAAGGGAGCGACTCT  TTCTCCTCCACACACAGAACGC  GCCGCCGTTGCTTGTTATTTGA  CTGCCACATCTTTGACGGCTCT  ATCGACTCCAAGAGGGCAAACG  GTCACCCGATTACCACCGACAG |
| *GhFIE-D*-RT-F | ATCGACTCCAAGAGGGCAAACG |
| *GhFIE-D*-RT-R | GTCACCCGATTACCACCGACTG |
| *GhMSI1-A*-RT-F  *GhMSI1-A*-RT-R  *GhMSI1-D*-RT-F  *GhMSI1-D*-RT-R  *GhEMF2-1A*-RT-F  *GhEMF2-1A*-RT-R  *GhEMF2-1D*-RT-F | GCCTTTGGATGGGGCTTGTAGT  AGAGCTTTGTTCTTGGGAGTAGCA  TTGGGACTTGAGAGCCCCTTCT  CCCATACCATGAGCCTTCTGCC  GTCTGGAAACAATCTTGCGCCC  GGCTGTGCTCTATGCGAGTGAA  TGTCATGGGAAAAAGCCCCAAACT |
| *GhEMF2-1D*-RT-R *GhEMF2-2A*-RT-F  *GhEMF2-2A*-RT-R  *GhEMF2-2D*-RT-F  *GhEMF2-2D*-RT-R  *GhVRN2-A*-RT-F  *GhVRN2-A*-RT-R  *GhVRN2-D*-RT-F  *GhVRN2-D*-RT-R | CCTTTGCCCCAACTTCTTCAGC  GAGGATGCTGCCTTTGGGGTAG  GAAGCTGCAGTGGTTGATTCGC  GTGGTGTTTCCTCGTCATCGCT  GCAAGTGATGCCTCAGACCCTT  AGAAAGCAAAGGGTCCTGGCTG  CAGTTGTTCATTGCGCCAGCAT  CTTCGTTGCCGTGCTTTACACA  TCAACCTCCTTTTACGCCTCGC |

| *GhUBQ7*-RT-R | GAAGGCATTCCACCTGACCAAC |
| --- | --- |
| *GhUBQ7*-RT-R | CTTGACCTTCTTCTTCTTGTGCTTG |

*****, Primers for qRT-PCR were designed on the qPCR Primer database (<https://biodb.swu.edu.cn/qprimerdb/>). The homologous genes derived from At- and Dt-subgenomes can not be distinguished in qRT-PCR assays because of their high similarity.
